# Consequences of chirality on the dynamics of a water-soluble supramolecular polymer

**DOI:** 10.1038/ncomms7234

**Published:** 2015-02-20

**Authors:** Matthew B. Baker, Lorenzo Albertazzi, Ilja K. Voets, Christianus M.A. Leenders, Anja R.A. Palmans, Giovanni M. Pavan, E.W. Meijer

**Affiliations:** 1 Institute for Complex Molecular Systems, Eindhoven University of Technology, 5612 AZ Eindhoven, The Netherlands; 2 Department of Innovative Technologies, University of Applied Sciences and Arts of Southern Switzerland, Galleria 2, CH-6928 Manno, Switzerland

## Abstract

The rational design of supramolecular polymers in water is imperative for their widespread use, but the design principles for these systems are not well understood. Herein, we employ a multi-scale (spatial and temporal) approach to differentiate two analogous water-soluble supramolecular polymers: one with and one without a stereogenic methyl. Initially aiming simply to understand the molecular behaviour of these systems in water, we find that while the fibres may look identical, the introduction of homochirality imparts a higher level of internal order to the supramolecular polymer. Although this increased order does not seem to affect the basic dimensions of the supramolecular fibres, the equilibrium dynamics of the polymers differ by almost an order of magnitude. This report represents the first observation of a structure/property relationship with regard to equilibrium dynamics in water-soluble supramolecular polymers.

## Abstract (web summary)

Water-soluble supramolecular polymers assemble from individual building blocks, but there is a lack of understanding as to how the properties depend on the components. Here the authors show how the introduction of chiral groups can affect the structural features and dynamic behaviour of the polymer.

## Graphical abstract

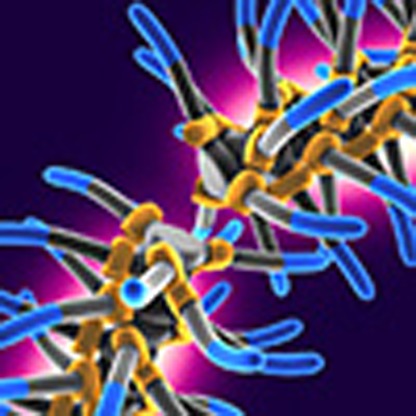

Supramolecular polymers, where monomers are not covalently linked, but are connected through non-covalent interactions, have seen recent emergence into functional materials^1^, often replacing traditional covalent polymers due to superior materials properties. Synthetic supramolecular polymers in aqueous solution are particularly attractive in foodstuffs, cosmetics and biomedical applications^2^, due to their dynamic and responsive nature. Systems based on peptide amphiphiles^3^,^4^, urediopyrimidinones^5^,^6^, cyclohexylbisureas^7^,^8^ and clay/cationic dendrimers^9^, to name a few, have all seen success from academic studies to industrial startups. While aqueous supramolecular systems have shown great promise, the design principles for one-dimensional supramolecular polymers in water is mostly empirical. A major challenge within the field of self-assembly lies in the ability to rationalize how small changes in the self-assembling structure can result in major or minor changes in the supramolecular assembly, akin to point mutations and their effect on protein structure/function. Commonly, small libraries of self-assembling molecules are made and their resultant supramolecular structures are evaluated based on mesoscale structural features or activity in an assay; the nanoscale differences and the molecular arrangement of monomers are often overlooked. Furthermore, the equilibrium dynamics of such systems, long touted as a benefit to the approach^10^,^11^,^12^, are sparsely investigated. While molecular mutations resulting in major structural changes are commonly documented in the field, the effect of structural changes on order and dynamics are much less clear. To further optimize the rational design of supramolecular systems, especially in water, it is imperative to investigate these details through a multi-scale approach (temporal and spatial).

We chose to use 1,3,5-benzenetricarboxamides (BTAs) as synthetic, water-soluble and one-dimensional supramolecular fibres. They are well known to self-assemble into supramolecular polymers via threefold hydrogen bonding and stacking of the BTA cores in a columnar helical fashion^13^. We have recently reported that the introduction of amphiphilic side arms (C_12_-PEG_4_-OH, Fig. 1a, 1) to the BTA core allows the reliable formation of thin, multiple micron length supramolecular fibres in water with a similar columnar arrangement of BTA units (Fig. 1b)^14^. Since then, these water-soluble BTAs have proven their utility as an aqueous supramolecular polymer and have been used to study superselectivity and spatiotemporal control of monomers^15^, to elucidate the mechanisms of monomer exchange^16^ and to form tailor-made hydrogels^17^.

During the initial study of these water-soluble BTA derivatives, both an achiral (**1**) and a chiral (**2**) BTA were synthesized, and aside from some striation seen in fibres of **1**, both molecules showed geometrically similar self-assembled fibres via cryo-transmission electron microscopy (TEM). However, the ultraviolet–visible absorption of the two fibres were starkly different (Fig. 1c), suggesting different arrangement of the aromatic cores, while circular dichroism spectroscopy showed that **2** created fibres of a preferred chirality^14^. Consequently, we were presented with a unique situation—two similar molecules (differing only by three stereogenic methyl groups) that produced similar supramolecular structures, despite measureable differences in the packing of the monomers. Surprised that these differences on the molecular level did not translate to the supramolecular structure, we wanted to investigate the origins and implications of this dichotomy.

Interested in the difference between the fibres from the molecular to the supramolecular scale, we have employed a variety of techniques, including small-angle X-ray scattering (SAXS), stochastic optical reconstruction microscopy (STORM), Förster resonance energy transfer (FRET) and molecular dynamics (MD) simulations to investigate the behaviour of the assemblies on multiple length and timescales. By thoroughly examining the differences between the achiral and chiral assemblies, we again document the ability of small molecular changes in assembling molecules to lead to large, and unpredictable, changes in the behaviour of the self-assembled structure. While the structures produced by the achiral and chiral BTAs look dimensionally similar, significantly different equilibrium dynamics are found between the two systems—an observation that has not been previously reported. In the absence of molecular level experimental detail, we turn to all-atom MD simulations to provide insight into the roots of this difference. We show that while molecular changes in the monomers do not greatly disturb the supramolecular structure, the addition of stereogenic methyls to the monomers noticeably increases both the internal molecular order and the persistence of hydrogen bonding within the simulated fibres.

## Results

### Molecular design and synthesis of monomers

**Figure 1:**
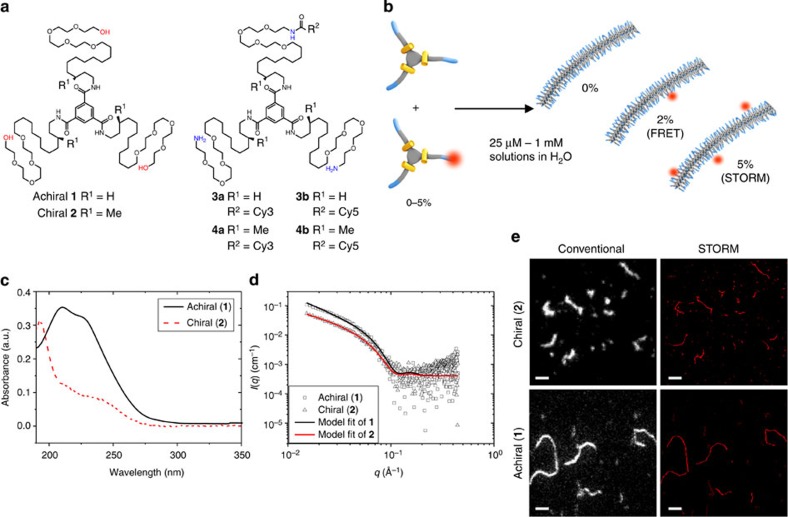
Overview of molecular and supramolecular structures used in this study. Shown are the molecular structures of an achiral (**1**) and chiral (**2**) BTA (**a**), both of which have been previously shown to self-assemble into high-aspect ratio fibres in aqueous solutions (**b**)^14^. Methodology to convert the terminal –OH in these structures to a terminal –NH_2_, label statistically with a dye, and separate the singly labelled compounds (**3a–b**, **4a–b**, see Supplementary Information for synthetic methodology) allows fluorescent labelling of these two supramolecular systems. Differences in the ultraviolet spectra of the two assembled fibres are seen when injected from MeOH into H_2_O (**c**, 10 μM concentration, 0.2 vol% MeOH, equilibrated for 24 h at room temperture); however, their characterization on the mesoscale is very similar. Experimental SAXS profiles (**d**) of achiral (**1**, 0.45 wt% in H_2_O) and chiral (**2**, 0.25 wt% in 96% H_2_O/4% MeOH), fit with the Schurtenberger–Pedersen form factor, yield *L*_K_=19.1±17.3 and 63.3±61.1 nm (**1** and **2**, respectively), *L*_c_>191 nm, and *r*_cs_=3.1±0.2 nm for the fibres. STORM (**e**, scale bar 2 μm) imaging shows both samples contain individual flexible fibres that are multiple microns in length.

The supramolecular monomers (both achiral **1** and chiral **2**) consist of a BTA core, an aliphatic carbon spacer and a tetraethylene glycol tail (C_12_-PEG_4_-OH or *(S)-*3-Me-C_12_-PEG_4_-OH, respectively, Fig. 1a). During the initial design of these molecules, it was hypothesized that the BTA units would provide directional self-assembly, the aliphatic spacer would shield the hydrogen bonds from water and provide a hydrophobic driving force (the absence of this spacer gave only molecularly dissolved species)^18^, while the ethylene glycol tail would impart water solubility to the structure. The chiral monomer (**2**) included homochiral methyl branches, designed to bias the helicity of the supramolecular fibre and enable further characterization and control over the supramolecular assemblies.

To gain insight into the differences between the two supramolecular systems (*vide supra*), monomers containing a fluorescent probe were desired to obtain fluorescently labelled fibres. Accordingly, BTA monomers containing a Cy3 or a Cy5 dye were synthesized (**3a**,**b** and **4a**,**b**), enabling the systems to be examined both by FRET exchange experiments^15^ and by super-resolution microscopy (STORM)^16^. We chose to conjugate the cyanine dyes via amide bond formation (amine on BTA and activated ester on the dye) since this chemistry is straightforward and low percentages of amine-terminated BTA monomers (based on **1**) have been previously shown to reliably incorporate into a BTA fibre^15^,^16^. Since previous synthetic strategies to produce a tris-amine analogue of BTA (**1**) involved long linear procedures (seven steps for achiral version, at least 14 steps for the chiral version), new methodology to convert the terminal hydroxyls of an intact BTA to terminal amines was developed. While a detailed synthetic discussion is outside of the scope of this manuscript, the synthetic procedures are reported and briefly discussed in the Supplementary Information (Supplementary Methods and Supplementary Figs 1–18). With the adoption of this new methodology, the production of milligram quantities of BTAs with synthetically addressable amines is now only three steps from the parent compound. With the tris-amines in hand, a sub-stoichiometric statistical coupling to a dye and separation of the singly labelled product was able to produce chiral **4a** and **4b** (or achiral **3a** and **3b**) in milligram quantities sufficient for all fluorescence based experiments.

### Indistinguishable mesoscale fibres

While individual fibres look similar via cryo-TEM, an ensemble measurement can nicely compliment this comparison. Shown in Fig. 1d, the SAXS profiles of the achiral (**1**) and chiral (**2**) BTAs are both characteristic for high aspect ratio, one-dimensional objects, with a contour length beyond the experimentally accessible range. Data analysis with a worm-like chain model yields a contour length (*L*_c_) greater than 191 nm, a Kuhn length (*L*_k_) of tens of nanometers and a cross-sectional radius (*r*_cs_) of 3.1 nm for both systems, which is in good agreement with the cryo-TEM images previously reported (Table 1). Consequently, both the achiral (**1**) and chiral (**2**) fibres are nearly indistinguishable by SAXS based on their cross-sectional area, stiffness or overall length. Furthermore, these results strongly suggest that both systems are single-column stacks of BTAs forming individual fibres in solution (no bundling or superstructure formation), even at the relatively high concentrations used in the SAXS measurements (≈0.5 wt%).

For further information on the shape and dimensions of individual fibres, we turned to super-resolution STORM imaging of achiral (**1**) and chiral (**2**) BTA fibres incorporating BTA-Cy5 monomers (**3b** and **4b**). STORM can probe structures adsorbed on glass from 25 nm to the multi-micron scale, therefore being complementary with *in situ* SAXS measurements (1–100 nm) and the vitrified cryo-TEM images (2–1,000 nm). Furthermore, we recently reported the ability of STORM microscopy to elucidate mechanistic and structural properties of synthetic supramolecular polymers^16^. The results of conventional, diffraction limited imaging (left) and STORM imaging (right) of chiral and achiral fibres is shown in Fig. 1e. Both samples show the presence of individual high aspect ratio fibres, with cross-sections below the resolution of the technique (<30 nm) and multi-micron contour lengths. By this technique, the fibres formed by the achiral (**1**) appear slightly longer than the chiral (**2**), but the magnitude of these differences are small when investigating the molecular scale. Furthermore, the chiral and achiral fibres show similar persistence length in the order of several hundreds of nanometers, a measurement that was outside of the range of SAXS (tens of nanometers).

### Significant differences in equilibrium dynamics

**Figure 2:**
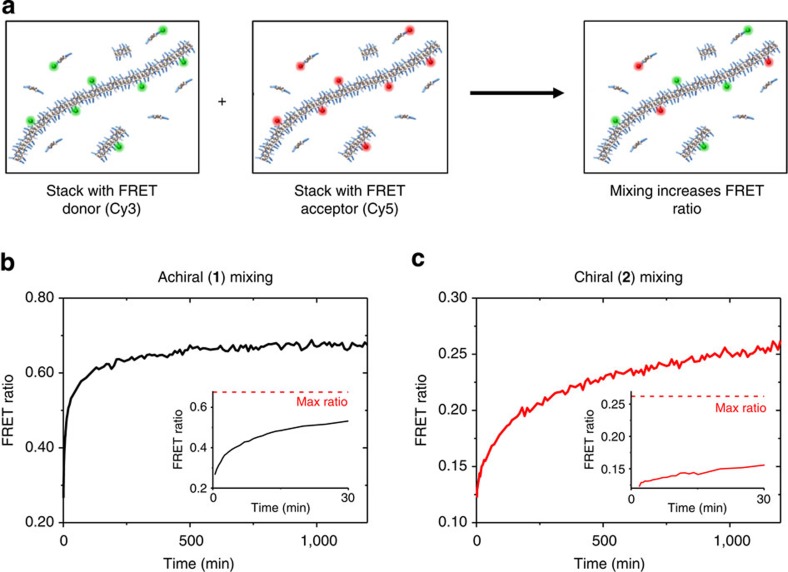
Exchange kinetics between both the achiral (1) and chiral (2) fibres. Assembling two BTA systems labelled with a donor (Cy3) or acceptor (Cy5) FRET pair, mixing the two samples, and measuring the FRET ratio in time gives information on the kinetics of exchange between the fibres in the two systems (**a**). Performed *in triplo*, the kinetics of exchange between two achiral fibres (**1**, **b**) is significantly faster than the kinetics of exchange between two chiral fibres (**2**, **c**) under identical conditions. It is hypothesized that this mixing occurs through weak points in the fibre, supported by the differences seen in the atomistic simulations (Fig. 4).

While characterization on the mesoscale shows very similar structural features for both the achiral (**1**) and chiral (**2**) systems, the TEM, SAXS and STORM measurements as discussed provide little information on the dynamics of the fibres. As a way to examine the monomer exchange of our supramolecular systems, we have previously introduced a FRET mixing-based assay^15^. Shown schematically in Fig. 2a, when two samples are separately co-assembled with a small amount (2–5%) of FRET paired dyes and then mixed, monitoring the change in FRET ratio over time gives the timescale for monomer exchange between the fibres. With this set-up, monomers trading between fibres increases the proximity of the FRET pairs, thereby increasing the FRET ratio, until a plateau is reached.

Seen in the FRET curves in Fig. 2b, the chiral (**2**) fibres exchanged monomers much slower than the achiral (**1**) fibres. Here we found that the achiral (**1**) system reaches a plateau in its FRET ratio after about 2 h, while the chiral (**2**) system has still not fully plateaued even after 20 h of equilibration. Furthermore, the exchange in both systems can be described using a biexponential process, with both the slow and fast timescales differing by almost an order of magnitude (raw data and fittings shown in Supplementary Figs 19–22). The physical origin of this biexponential behaviour is unclear; however, it does suggest that the two systems exchange monomers in a similar fashion.

Previously, STORM images with two populations of differently coloured fibres have been used to clearly elucidate that exchange between achiral (**1**) BTA fibres occurs randomly along the length of the fibres^16^. However, the chiral (**2**) poorly adhered to the glass slides and prevented the time-lapsed imaging of single fibres during the exchange process. This result emphasizes the fine balance and optimization of surface chemistry required for reliable sample preparation during STORM imaging, currently an active area of research.

At this point, it becomes clear that although the fibres appeared the same, the addition of a stereogenic methyls to this supramolecular system greatly reduces the kinetics of exchange between the fibres. The addition of three homochiral methyl groups does make chiral **2** more apolar, and hence slightly more hydrophobic, but the magnitude of difference is surprising. In addition, seeing a difference in the molecular arrangement of the BTA cores in the two fibres, as indicated by ultraviolet–visible spectroscopy, we aim for a more detailed molecular level picture of the fibres.

### Relaxation dynamics using all-atom MD simulations

Herein, we employed all-atom MD simulations, proven an effective technique to observe molecular order and dynamics of supramolecular systems^19^,^20^,^21^,^22^. While significant computational effort has been given to BTA assemblies in the gas phase^23^,^24^,^25^, and organic solvents^26^, very little atomistic-level work has been performed on any synthetic supramolecular fibres in water^20^, aside from peptide amphiphile structures^27^,^28^,^29^.

**Figure 3:**
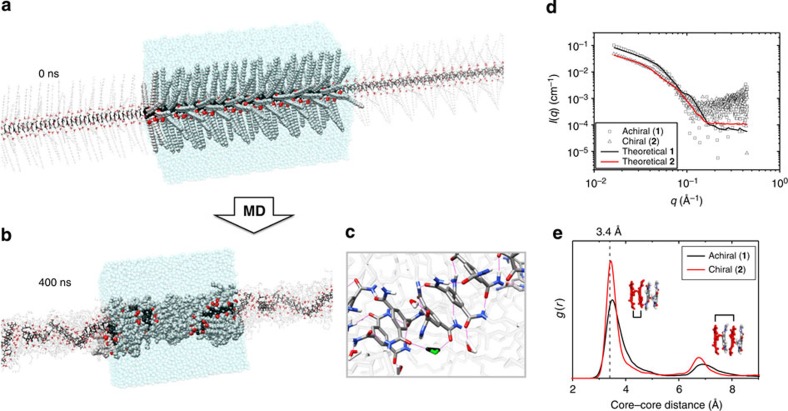
Overview of simulation results from achiral (1) and chiral (2) fibres. Starting from a geometry of gas-phase optimized cores, with extended side chains, the achiral (**1**), (**a**) chiral (**2**) and discordant (2*) simulated systems were equilibrated in water for 400 ns. (**b**) Equilibrated (last) configuration of the chiral (**2**) fibre taken from the MD simulation: BTA cores are coloured in black, methyl groups in red and BTA side chains in grey, water molecules are shown in the simulation box (transparent cyan) that is replicated in space producing an infinite fibre. (**c**) Despite the folding of the side chains to screen the BTA cores from contact with the external solution, water molecules are able to penetrate into the fibre and to interact with the hydrogen bonding motif of the BTA cores (single bridging water molecule highlighted in green and black). (**d**) As a measure of fidelity of the calculations, the final equilibrated structures were able to accurately reproduce (black and red line) the experimentally determined SAXS profiles for the fibres (circles and squares). (**e**) Radial distribution functions *g(r)* of the neighbour BTA cores along the fibres calculated from the equilibrated phase MD trajectories (the last 100 ns). Simulations start from an initial configuration with intercore spacing of 3.40 Å (dashed line). The relative height and position of the *g(r)* peaks provide information on the order in the stacking between BTA neighbours in the equilibrated fibres (Supplementary Figs 27 and 28).

Given the size and the complexity of the system to be studied, capturing the polymerization of individual BTAs into a fiber at atomistic resolution in water is unfeasible; consequently, we decided to start from an ideally arranged supramolecular structure and to further relax the fibres. As a first step, we built atomistic models for the achiral (**1**) and chiral (**2**) fibres starting from 48 self-assembled, prestacked cores, previously optimized with gas-phase density functional theory calculations^24^. Extended lateral chains for achiral (**1**) and chiral (**2**) (C_12_-PEG_4_-OH or *(R)-*3-Me-C_12_-PEG_4_-OH, respectively) were added to the starting geometry to obtain the initial configuration for the fibres as seen in Fig. 3a. Initially, the extended fibres are characterized by an intercore (between BTAs) distance of 3.4 Å, a starting diameter of 6.8 nm and an initial amide bond dihedral angle of −140° (C_AR_–C_AR_–C_=O_–O_=C_), consistent with a *P*-helicity. Previous studies have shown the *S* enantiomer of a chiral water-soluble BTA prefers the *M*-helicity; thus, the sidearms for chiral (**2**) were constructed with *R* stereogenic methyls^30^,^31^. Since both fibres **1** and **2** were built up from idealized structures, we also created a fibre with *P*-helicity and *S* stereogenic methyls, where the helicity of the fibre and the chirality of the monomer does not match the experiment. This fibre was constructed as additional comparison, aiming at capturing the effects of the chiral bias as seen in the experimental circular dichroism spectra (*vide supra*).

During the early steps of the simulations, the side chains of the BTAs collapse around the core of the fibre to reduce the amount of hydrophobic area exposed to the solvent (primary folding). However, significant voids remain where water is in direct contact with the hydrophobic core of the fibres. Consequently, the initially linear fibres also begin to fold (secondary folding), further reducing the exposed hydrophobic area. Owing to this secondary folding, the length of the simulated fibre (48 BTAs) changes from an initial value of 16.3 nm, for both (**1**) and (**2**), to final values of ≈7 nm (**1**) and ≈10 nm (**2**) during the MD simulation (Supplementary Movies 1–3). Both fibres approached an equilibrium after ≈300 ns; various parameters used to monitor the equilibration showed good convergence during the last 100 ns of MD (Supplementary Figs 23–25)—this phase was thus used for further analyses as representative of the equilibrium (Fig. 3b shows the chiral (**2**) fibre at the end (400 ns) of the MD simulation).

To compare the equilibrated structures to experimental results, theoretical SAXS profiles were generated from the PDB files of the equilibrated fibres using CRYSOL from the ATSAS software package^32^. Satisfyingly, the theoretical profiles match nicely with the experimental results obtained (Fig. 3d), showing the ability of our simulations to approximate the structural characteristics of a BTA fibre in solution. Furthermore, to demonstrate that our model parameterization was capable of capturing the spontaneous self-assembly of the BTA molecules, seven molecularly dissolved and monodisperse BTAs, prefolded in water, were placed in a simulation box and allowed to equilibrate under MD conditions identical to the fibre simulations (400 ns). Self-assembly was observed, and this simulation showed the formation of a stable, stacked BTA trimer formed after ≈150 ns of simulation (Supplementary Fig. 26 and Movie 4). The consistency of the MD simulation with X-ray structural data and its ability to capture spontaneous BTA self-assembly in solution supported the reliability of the molecular models and promoted confidence in further molecular level analysis of the self-assembled structures.

*Chiral BTA fibres exhibit a higher level of internal order*. The introduction of helicity is known to impart a higher level of order in the columnar assemblies in the gas phase^33^,^34^; however, little is known about the directing effect of helicity in aqueous environments. While the organic solution and gas-phase geometries of BTA stacks show regularly packed and ordered cores, distortions from this order are necessary to accommodate the hydrophobically driven folding and bending of the fibre in water (Supplementary Figs 27 and 28). Seen in Fig. 3e, radial distribution functions (*g(r)*) of the intercore distances between BTAs during the equilibrated phase of the MD simulations (the last 100 ns) show different peak heights and widths at the characteristic BTA stacking distance (≈3.4 Å, dashed line)^23^,^24^,^26^,^35^. As the *g(r)* measures the probability of finding neighbouring BTA cores at variance of the distance, the data suggest that the stacking order of the aromatic cores is on an average higher within the chiral (**2**) than in the achiral (**1**) fibre by ≈+52%.

**Figure 4:**
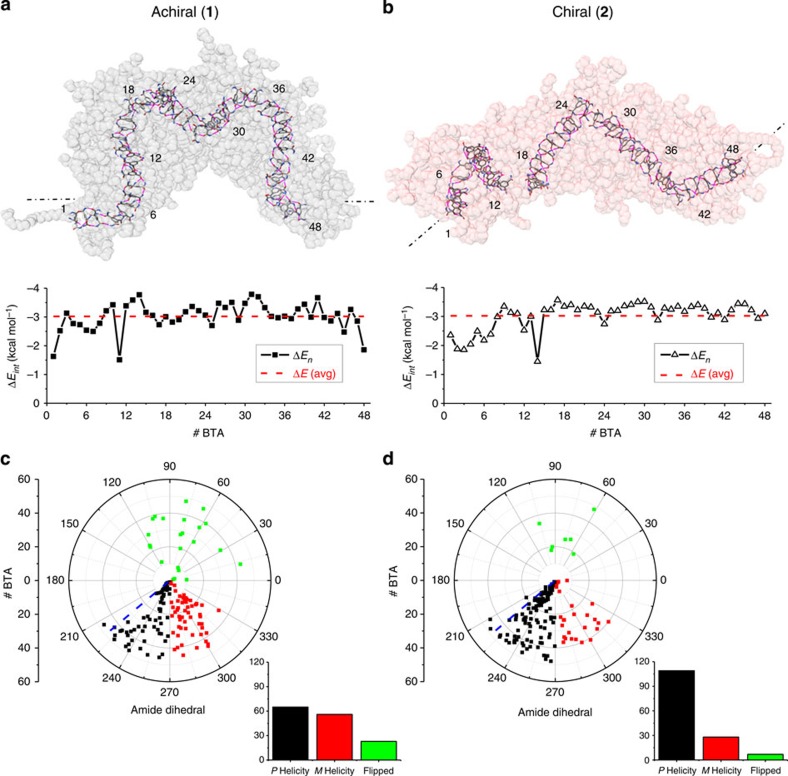
Energetics and order in the achiral (1) and chiral (2) equilibrated fibres. (**a**,**b**) Fibres (last MD snapshots) are shown with transparent side arms, BTA cores are coloured per atom (N: blue, O: red, C: grey, H: white), and hydrogen bonds are coloured in pink. Self-assembly interaction energy (Δ*E*) of individual BTA molecules. Δ*E* are expressed in kcal mol^−1^ and accounts for solute–solute and solute–solvent interaction of an individual BTA with the global system. While in the achiral (**1**) system order/disorder is uniform along fibre, the chiral (**2**) fibre is composed of stable domains (≈7 BTAs), with consecutive Δ*E* values more favourable (negative) then the average one, separated by unstable (weak) points. (**c**,**d**) Further differences can be seen in the dihedral angles (C_AR_–C_AR_–C_=O_–O_=C_) between the achiral (**1**) and chiral (**2**) systems. Averaged over the last 100 ns of simulation, the dihedral angles of the achiral sample (**1**) are more disordered and more likely to flip over the plane of the benzene ring (see also Table 1), as compared with the chiral system (**2**). In all samples, the dihedral angles are more out of the plane of the aromatic ring when compared to the previously simulated structures^23^,^24^,^25^,^26^ (**c**,**d**, blue dashed line).

**Figure 5:**
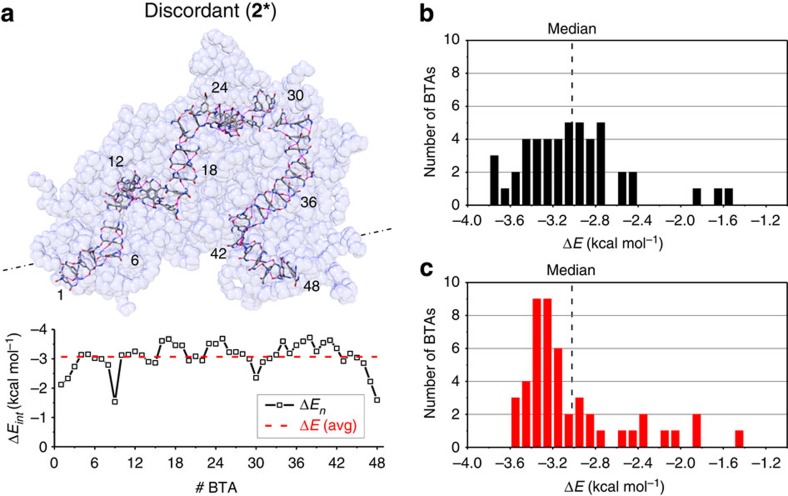
Order in the (2*) fibre and energetic distributions of achiral (1) and chiral (2). The simulation of the additional system composed of 48 (2*) BTAs starting from the same *P-*helicity configuration of (**1**) and (**2**), but having reverse methyl initial displacement, helps to decompose the effect of chirality and of the steric stabilization introduced by methyl branching on the fibre order. (**a**) Self-assembly interaction Δ*E* energy of the (2*) BTAs in the fibre—no presence of long BTA stable domains is identified as in (**2**). (**b**,**c**) Histograms for the number of BTAs in the fibres with given Δ*E* energy (**b**, achiral (**1**); **c**, chiral (**2**)). Even if the three systems do not differ in terms of median Δ*E* value (=−3 kcal mol^−1^, dashed lines) the distribution of the Δ*E* energy values around the median are different (same data for (2*) in the SI). In particular, the achiral (**1**) system shows a nearly Gaussian distribution of Δ*E* around the median, while the chiral (**2**) is characterized by higher number of stable stacked domains (those with more negative Δ*E* than the average value).

To assess differences in the BTA–BTA interactions within the fibre, we extracted the self-assembly energy (Δ*E*) of individual BTAs in the systems. This energetic term (Δ*E*) accounts for the average interaction energy of a BTA with all other BTAs in the fibre, or the energy gain for one BTA to stay incorporated with the fibre rather than being molecularly dissolved in solution (negative Δ*E* values indicate favourable interactions). Surprisingly, the average self-assembly energies in the two fibres are similar, suggesting that one fibre is not particularly more stable than the other. However, when one looks at how uniformly the Δ*E* values are distributed between all BTAs along the length of the fibre, differences emerge (Fig. 4). The achiral (**1**) fibre shows that the Δ*E*_*n*_ values for the individual BTAs vary randomly above and below this average when travelling along the fibre (Fig. 4a). Meanwhile, the chiral (**2**) fibre shows runs of stable BTAs (stable self-assembled domains) within the fibre with Δ*E*_*n*_ below average (5–7 BTAs: for example, BTA 25–31, Fig. 4b) interrupted with unstable points (for example, BTA 13) with Δ*E*_*n*_ above average. Furthermore, histograms of the same data (Fig. 5b,c) show the achiral (**1**) fibre has a nearly Gaussian distribution of the individual BTA energies around the average, while the chiral (**2**) fibre shows a distribution that is significantly skewed by unstable BTAs within the fibre, though most are more stable than the average (stable domains).

Owing to the importance of hydrogen bonding for the assembly of BTA fibres, the dihedral angles of the amide bonds and the hydrogen bonding motif play a major role on the properties of an assembly. Since both fibres started from the same initial structure (amide dihedrals of −140°, all with *P*-helicity), it was possible to track their evolution during the MD runs. In both fibres, the average dihedral angle (during the last 100 ns) is more out of the plane of the aromatic ring as compared with the gas-phase starting geometry (Fig. 4c,d)^23^,^24^ or MD simulations in an organic solution^26^. However, as expected, the chiral BTA (**2**) better preserves the dihedrals of the amide bonds (and thereby the chirality of the assembly) in the initial *P*-helicity (between −90° and −180°, 76% average a *P*-helicity), while the lack of chiral information in the achiral BTA (**1**) leads to an almost equal distribution of dihedrals in both *P*- and *M*-helicity (45 and 39%, respectively). In addition to this preservation of helicity, there is also a significant difference between the number of dihedrals that flip over the aromatic core in the two fibres (green data points in Fig. 4c,d, positive dihedral angles). Only seven dihedrals (5%) have positive angular values in the chiral (**2**) fibre, while the achiral (**1**) fibre contains 23 flipped dihedrals (16%). This suggests that not only does the presence of the stereogenic methyls in **2** influence the chirality of the hydrogen bonding motif, but it also hinders the ability of the amide dihedrals to invert over the plane of the aromatic ring.

*Hydrogen bonding and hydrophobicity*. At this point, a major question emerges: is the behaviour of these fibres controlled more by hydrogen bonding (as it is the case in organic solvent)^13^ or by hydrophobic effects? The interplay between the two components is crucial (as seen in self-assembled peptidic structures)^27^,^36^, but the dominant factor is often unclear.

Seen extensively in BTA assemblies in organic solution, we have also observed a similar hydrogen bonding motif in aqueous assemblies of **1** via infrared measurements in D_2_O (Supplementary Fig. 35). In line with this experimental evidence, the simulations of both the achiral (**1**) and chiral (**2**) fibres show the presence of hydrogen bonding throughout the structure; however, there are significant deviations from the hydrogen bonding seen in apolar organic solutions. Although the fibre folds dramatically to reduce the exposed hydrophobic surface area, significant penetration of water into the hydrophobic core is still apparent; shown in Fig. 3c, water molecules are clearly able to interact with, and eventually break up, the hydrogen bonds between adjacent BTAs (see Supplementary Figs 29 and 30 for radial distribution functions of water molecules in the interior of the fibres). The increased molecular order in the chiral (**2**) fibre can also be seen in the persistence of the hydrogen bonding motif within the fibre during the simulations. The number of persistent hydrogen bonds (those present more than 95% of the time: 39 for **1**, 56 for **2**) between the BTA cores fits well with the differences in the order of BTA cores between the two fibres (Table 2).

Analysis of the average pairwise interaction energy for each BTA with other individual BTAs in the equilibrated fibres allows us to determine the major driving forces for the self-assembled structures in an aqueous environment. Naturally, BTAs interact more strongly with their closest neighbours (high favourable Δ*E*_int_ values), while the pairwise interaction becomes smaller for farther BTAs; after about eight neighbours, the interaction of the BTAs with the fibre matrix saturates, consistent with their incorporation in a bulk environment (see Supplementary Figs 31 and 32 for details). Shown in Table 3, the average pairwise interaction energy of BTA with the closest neighbour monomers (Δ*E*_int_, accounting for solute–solute and solute–solvent interactions) are nearly identical between the achiral (**1**) and chiral (**2**) fibres (−38 and −35 kcal mol^−1^, respectively) consistent with the similar energetics between the two systems. Interestingly, the solvation terms of Δ*E*_int_ (Δ*E*_int,sol_) are similar between the two systems (−10.8 (**1**) and −10.0 (**2**) kcal mol^−1^), suggesting that the presence of the stereogenic methyls does not significantly change the solvation energies of the fibres. When analysing the contributions to this interaction, one quickly sees that, in terms of the interaction between neighbours, the electrostatic contribution (capturing H-bonding) is approximately half of the solvation contribution (capturing hydrophobic effects). While the supramolecular polymerization of BTAs in organic solvents is driven by strong cooperative hydrogen bonding between BTA monomers, analysis of these simulations clearly show that hydrophobicity dominates any electrostatic interactions (including hydrogen bonding) in water.

The contribution due to the hydrogen bonding motif (Δ*E*_HBs_) can be directly extracted from the number of persistent hydrogen bonds per-BTA (Table 3), taken along with the average energy per single hydrogen bond in aqueous solution for peptidic structures (≈−1.58 kcal mol^−1^)^37^. For example, in the achiral (**1**) fibre, we can estimate the contribution of hydrogen bonding to BTA self-assembly is −2.6 kcal mol^−1^. While hydrogen bonding pertains to the closest BTA neighbours in the fibre, hydrophobicity is a long-range effect; in this way, when accounting for all BTAs in the fibre in the calculation of the solvation energy, Δ*E*_HBs_ is not even 7% of the global energy of solvation (Table 3: Δ*E*_sol_≈−40 kcal mol^−1^ for the whole fibres). Even accounting for cooperative self-assembly^24^, the energy of solvation (hydrophobic effect) still dominates hydrogen bonding. Performing this same analysis on the chiral fibre gives more importance to the hydrogen bonds (mainly because there are more present); however, the energy of solvation still outweighs the energy of hydrogen bonding by an order of magnitude.

*Does chirality stabilize the fibres.* As it is clear that the stereogenic methyl groups in chiral (**2**) increase fibre persistency and reinforce the hydrogen bonding network, as compared with (**1**), this result opens a second major question: is this the effect of the reinforcing chirality or simply a steric bias introduced by the presence of the branched methyls? And in this framework, if we started a simulation with a fibre where the helicity of the stack and the chirality of the monomer are discordant (the all *S* enantiomer, denoted discordant 2*) from our experimental observations, what happens?

Seen in Fig. 5, the discordant (2*) fibre equilibrates qualitatively similar to those of achiral (**1**) and chiral (**2**), whereby the alkyl chains collapse (primary folding) and the fibre folds decreasing the interactions with water (secondary folding). Interestingly, the shortening and distortion of the discordant (2*) fibre (≈7.2 nm final length) is nearly identical to that of the achiral (**1**) fibre, and in general more pronounced than for the chiral (**2**) fibre (Table 2, Supplementary Figs 24 and 25). In addition, the resulting arrangement of the cores in the equilibrated (2*) again shows uniform disorder (Fig. 5a), reminiscent of the achiral (**1**) fibre and no presence of long stable BTA domains as seen for the chiral (**2**) fibre.

In the equilibrated phase of the MD simulation (the last 100 ns), the discordant (2*) fibre contains amides in both the *P-* and *M-* helicity (58% and 35%, respectively). Interestingly, while in the achiral (**1**) fibre, the initial chirality was nearly lost during the simulation (equal amounts of amides in *P*- and *M*- helicity), the discordant (2*) fibre still has an overall *P-*helicity (Supplementary Fig. 33). This result suggests that the presence of a stereogenic methyl increases the barrier height to helix inversion, even when in an incorrect geometry.

Furthermore, the addition of the branched methyl increases hydrogen bond stabilization where the number of persistent H-bonds in (2*) is intermediate between the achiral (**1**) and chiral (**2**). The energetic histogram (Supplementary Fig. 34) also confirms an intermediate behaviour of (2*), with reduced stable stacking domains as compared to **2**, but a less uniform distribution of disorder along the fibre compared with **1**. Data in Table 3 show that, in general, the presence of methyl groups in the BTA structure supports the stabilization of the hydrogen bonding network, albeit to a lower extent when chiral information is discordant. On the basis of these results, the observation of increased order and kinetic stability of the chiral (**2**) fibre is largely due to the homochiral nature of the fibre.

## Discussion

Within this study, both the achiral (**1**) and chiral (**2**) BTAs formed fibres that share many nearly identical structural and energetic features. They both form thin (radius ≈3.1 nm), long (>2 μm) fibres of appreciable persistence length that look nearly identical by cryo-TEM, SAXS and STORM. On the basis of simulation, they both form fibres that are more folded and disordered than the BTA fibres characterized in organic solution and the gas phase. From this, one quickly sees that hydrophobic interactions dominate the assemblies, while hydrogen bonding provides directionality to the structures. The chiral (**2**) fibres exchange monomers at much slower rate than the achiral (**1**) fibres and MD simulations show a significant difference in internal order between the two systems, suggesting this is not simply a difference in solubility. In MD simulation, the chiral (**2**) fibre showed a higher level of order in the stacking of the BTA cores (≈+52%), overall higher regularity including stable regions of highly ordered BTAs (≈7), and more persistence in the hydrogen bonding network (≈+44%). Furthermore, the simulation of a discordant fibre (2*) supported the observations seen during the other MD simulations, further suggesting that these observations are not simply due to the presence of branching within the structure.

During the MD simulations, the side chains of all modelled structures collapse around the core, followed by noticeable secondary folding of the fibre to further shield the hydrophobic core and causing the overall fibre length to shorten significantly from the initial structure (≈56% for achiral (**1**), ≈42% for chiral (**2**), and ≈56% for discordant (2*)). Despite this hydrophobically driven folding, the side chains cannot completely insulate the cores and water molecules can interact with, and disturb, the hydrogen bonding motif of the BTAs (Fig. 3c). Breaking down the energy contributions for the stabilization of one BTA by its neighbours (Δ*E*_int_), we consistently found that the stabilization energy of solvation was higher than that of hydrogen bonding. While competitive hydrogen bonding solvents can easily break up BTA assemblies in organic media, in aqueous solution, hydrophobic interactions are clearly more important to the integrity of the assembly; while hydrogen bonding does give directional order to the system, it is not the linchpin of the assembly.

In a previous publication, the exchange mechanism between achiral (**1**) fibres was probed with STORM and determined to proceed through random exchange along the fibres (not from the ends and not via breakage and recombination) of monomers, or groups of monomers through weak, disordered sections of the fibre.^16^ In this study, we have observed, through all-atom MD simulations, the distribution of disorder throughout both fibres and identified a lower number, but more pronounced unstable points within the chiral (**2**) system compared with achiral (**1**) system. If these regions are considered as weak points that can produce local fibre breakage and repair, these data propose a picture where the chiral fibres of (**2**) are more brittle and more likely to exchange larger groups of monomers (≈7 BTA, Fig. 4b) than the achiral (**1**), with a larger overall energy penalty for the process. In a scenario where BTAs exchange between fibres through (i) a first separation of groups of BTA monomers from one fibre, (ii) BTA diffusion in solution and (iii) BTA incorporation into another fibre, the data from MD well rationalize the hypothesis from previous STORM experiments and match nicely with the slower FRET exchange dynamics found for the chiral (**2**) system.

While the addition or subtraction of a chiral methyl may appear, at first, to have little consequence on the supramolecular structure, our BTA-based supramolecular polymer shows significant changes in internal order and dynamics based on the introduced homochirality. Analysis of the amides, hydrogen bonds and intercore spacing within the simulated structures show that, although branching has some positive effect on the persistence of the hydrogen bonds, chirality produces a significantly higher internally ordered supramolecular polymer. Greater order in the system results in a supramolecular fibre that maintains the shape of its achiral counterpart, but shows significantly reduced exchange dynamics. In addition, the presence of the methyl groups also appears to impart a certain level of brittleness to the internal structure of the fibre, consistent with our observation of qualitatively shorter fibre lengths via STORM microscopy. Further investigation of this embrittlement may provide a link to biomolecular assemblies.

From our studies, it appears that the use of stereogenic substitutions can provide a reliable way to rationally tune the kinetic stability of supramolecular fibres. Although much more work needs to be performed towards this goal, it is a promising design principle and the first observation of its kind.

The dynamic and responsive nature of supramolecular systems has promised to be their major benefit over traditional covalent polymeric systems. Although we have shown that the structure/dynamics/property relationships within an aqueous supramolecular polymer requires extensive characterization to elucidate, it is our hope that future attention to the molecular arrangement and dynamics of such supramolecular systems can rapidly facilitate the rational design of functional systems with tailor-made supramolecular structure, molecular arrangement and dynamics to match the application. To unlock the full potential of supramolecular systems, it is imperative that we begin to focus on the structure/dynamics/property relationships of the self-assembling monomers to facilitate major breakthroughs in self-assembled systems.

## Methods

### General

Unless stated otherwise, all reagents and chemicals were obtained from commercial sources at the highest purity available and used without further purification. Cy3- and Cy5-NHS esters were obtained from Lumiprobe. All solvents were of AR quality and purchased from Biosolve. Water was purified on an EMD Milipore Mili-Q Integral Water Purification System. Reactions were followed by thin-layer chromatography (precoated 0.25 mm, 60-F254 silica gel plates from Merck), and flash chromatography was run with silica gel (40–63 μm, 60 Å from Screening Devices b.v.).

^1^H-NMR and ^13^C-NMR spectra were recorded on a Varian Mercury Vx 400 MHz (100 MHz for ^13^C) NMR spectrometer. Chemical shifts are given in p.p.m. (*δ*) values relative to residual solvent. Splitting patterns are labelled as s, singlet; d, doublet; dd, double doublet; t, triplet; q, quartet; quin, quintet; m, multiplet and b stands for broad. Matrix-assisted laser desorption/ionization mass spectra (MALDI) were obtained on a PerSeptive Biosystems Voyager DE-PRO spectrometer or a Bruker autoflex speed spectrometer using α-cyano-4-hydroxycinnamic acid (CHCA) and 2-[(2E)-3-(4-tert-butylphenyl)-2-methylprop-2-enylidene]malononitrile as matrices.

Ultraviolet–visible absorbance spectra were recorded on and a Jasco V-650 UV–vis spectrometer with a Jasco ETCT-762 temperature controller. Fluorescence data were recorded on a Varian Cary Eclipse fluorescence spectrometer equipped with a sample changer and a peltier.

Preparative reversed-phase high-pressure liquid chromatography (prep-RP-HPLC) was performed on a system consisting of the following components: Shimadzu LC-8A preparative liquid chromatography pumps (with an Alltima C18 5 u (125 × 20 mm) preparative reversed-phase column and gradients of water–acetonitrile, supplemented with 0.1% trifluoroacetic acid), a Shimadzu CBM-20A prominence communications bus module and Shimadzu DGU 20A3 prominence degasser, Thermo Finnigan Surveyor PDA detector, Finigan LCQ Deca XP and Thermo Finnigan surveyor auto sampler.

Reversed-phase high-pressure liquid chromatography–mass spectrometry (RP-HPLC-MS) was performed on a system consisting of the following components: Shimadzu SCL-10 A VP system controller with Shimadzu LC-10AD VP liquid chromatography pumps (with an Alltima C18 3 u (50 × 2.1 mm) reversed-phase column and gradients of water–acetonitrile supplemented with 0.1% formic acid, a Shimadzu DGU 20A3 prominence degasser, a Thermo Finnigan surveyor auto sampler, a Thermo Finnigan surveyor PDA detector and a Thermo Scientific LCQ Fleet.

### Synthesis of monomers

Both the achiral (**1**) and the chiral (**2**) BTAs were synthesized according to previously published methodology^14^. Synthetic methodology for both the achiral and chiral tris-amines (**S5** and **S6**, respectively) are presented in detail in the Supplementary Information (Supplementary Methods). It should be noted that the transformations of **1** to **3** and **2** to **4** involve reliable high-yielding transformations of general interest to the macromolecular community; however, in light of brevity, only the final transformation is presented within the article and discussion of the synthesis is reserved for the SI. Also, the full characterization and spectra of all compounds is presented in the Supplementary Information (Supplementary Figs 1–18).

Representative procedure for dye labelling of monomers: in a 1.5-ml vial, tri-amine BTA (**S5**, 10 mg, 7.8 μmol) was dissolved into 0.5 ml of dry dimethylsulphoxide (DMSO) and a drop (≈20 μl) of TEA was added, followed by a solution of Cy3-NHS ester in dry DMSO (4.59 mg, 7.8 μmol, 1.0 equiv., 0.5 ml solution) and the reaction mixture was allowed to stir overnight. The next day, the reaction mixture was dialysed against water for 3 h to remove most of the DMSO (1000 MWCO membrane) and then the mixture was purified via preparative LC/MS to isolate only the monocoupled product (gradient from 50–70% MeCN). After purification and lyophilization, compound **3a** was isolated as a red amorphous film (3.3 mg, 1.6 μmol, 20% yield, calculated for tri-TFA salt). LC/MS of the product revealed a single peak corresponding to the expected mass (Supplementary Fig. 15). MS (ESI, *m/z*) calcd. for [M]^+^ C_99_H_167_N_8_O_16_, 1725.25; found, 1724.82.

**3b**: synthesized from **S5** and isolated as a blue film, 1.5 mg, 0.84 μmol, 11% yield. LC/MS of the product revealed a single peak corresponding to the expected mass (Supplementary Fig. 16). MS (ESI, *m/z*) [M]^+^ calcd. for C_101_H_169_N_8_O_16_,=1751.27; found, [M]^+^=1751.73.

**4a**: synthesized from **S6** and isolated as a red film, 0.8 mg, 0.44 μmol, 20% yield. LC/MS of the product revealed a single peak corresponding to the expected mass (Supplementary Fig. 17). MS (ESI, *m/z*) [M]^+^ calcd. for C_102_H_173_N_8_O_16_, 1767.30; found, 1767.82.

**4b**: synthesized from **S6** and isolated as a blue film, 0.75 mg, 0.41 μmol, 18% yield. LC/MS of the product revealed a single peak corresponding to the expected mass (Supplementary Fig. 18). MS (ESI, *m/z*) [M]^+^ calcd. for C_104_H_175_N_8_O_16_, 1793.32; found, 1793.82

### Assembly of samples

The assembly of supramolecular polymer samples was achieved through a dilution protocol (Fig. 1b). Stock solutions of BTA (**1** or **2**, 10 mM in MeOH) and dye labelled BTA (**3a**, **3b**, **4a**, and **4b**, ≈1 mM in MeOH) were prepared, and the stock solutions of dye labelled BTAs were standardized based on the absorbance of free dye in MeOH. These stock solutions were then combined and mixed to provide the correct concentration and dye ratio for the desired sample and diluted with filtered Milli-Q water. A typical preparation involved mixing 3.75 μl of BTA solution with 2 μl of dye labelled BTA solution, followed by dilution with 1,500 μl of water (producing 25 μM BTA concentration with 2% dye labelling). The samples were then allowed to equilibrate for 24 h before experiments, crucial for reliable stack formation. For FRET measurements dye labelling of 2% was used, while for STORM experiments 5% dye labelling was found to be optimal.

### Small-angle X-ray scattering

SAXS measurements were performed on a SAXSLAB GANESHA 300 XL SAXS system equipped with a GeniX 3D Cu Ultra Low Divergence micro focus sealed tube source producing X-rays with a wavelength *λ*=1.54 Å at a flux of 1 × 10^8^ photons s^−1^ and a Pilatus 300 K silicon pixel detector with 487 × 619 pixels of 172 μm^2^ in size placed at a sample-to-detector distance of 713 mm, respectively, to access a *q*-range of 0.15≤*q*≤4.47 nm^−1^ with *q*=4*π*/*λ*(sin *θ*/2). Silver behenate was used for calibration of the beam centre and the *q* range. Samples were contained in 2 mm quartz capillaries (Hilgenberg, Germany).

The two-dimensional SAXS patterns were brought to an absolute intensity scale using the calibrated detector response function, known sample-to-detector distance, measured incident and transmitted beam intensities, and azimuthally averaged to obtain one-dimensional SAXS profiles. The scattering curves of the supramolecular polymers were obtained by subtraction of the scattering contribution of the solvent and quartz cell. Finally, the absolute calibration of the scattering curves was verified using the known scattering cross-section per unit sample volume, dΣ/dΩ, of water, which is 0.01632, cm^−1^ at *T*=20 °C.

The SAXS profiles were analysed by two independent methods using the software packages SasView ( http://www.sasview.org/) and CRYSOL^32^. First, we selected a form factor developed originally for semi-flexible, self-avoiding polymer chains by Pedersen and Schurtenberger^38^ to which we refer as the worm-like chain model. This describes the scattering profiles of the supramolecular chains in terms of a contour length, *L*_c_, a Kuhn length, *L*_k_, and a cross-sectional radius, *r*_cs_. Next, the scattering profiles were compared with the theoretical scattering curves generated by the CRYSOL for the simulation results described (the PDB file used for generation of theoretical SAXS data was constructed from 10 simulation boxes arranged to form a portion of the infinite fibre, 480 BTAs).

### Stochastic optical reconstruction microscopy

STORM imaging was performed as previously described^16^. BTA fibres were immobilized by adsorption onto the surface of a flow chamber assembled from a glass slide and a coverslip separated by double-sided tape. Glass microscope coverslips (thickness 0.17 mm) were washed as previously decribed^16^. Images were acquired using a Nikon N-STORM system configured for TIRF imaging. Cy5-labelled samples were illuminated by the 647-nm laser lines built into the microscope. Fluorescence was collected by means of a Nikon × 100, 1.4 NA oil immersion objective and passed through a quad-band-pass dichroic filter (97335 Nikon). Frames (30,000) were recorded onto a 128 × 128 pixel region of an EMCCD camera (ixon3, Andor). STORM images are reconstructed with the STORM module of the NIS element Nikon software.

### FRET mixing experiments

For FRET mixing experiments, two samples of supramolecular fibres labelled with either a Cy5 or a Cy3 were mixed in a 1:1 ratio in a 500-μl cuvette and immediately measurements were started. To determine the FRET ratio, samples were excited at 520 nm (Cy3 excitation, donor) and monitored at both 570 nm (Cy5 emission, donor) and 670 nm (Cy5 emission, acceptor). Temperature was kept at 20 °C using the instrument’s peltier. Experiments were run at least *in triplo* to ensure reproducibility of the results. Curves were fit with a biexponential process and details of the fitting are presented in the Supplementary Information (Supplementary Figs 19–22 and Tables 1, 2).

### MD simulation

The entire simulation work was conducted using the AMBER 12 software^39^. The molecular models for the BTA monomers used in the MD simulations were built and parametrized according to a validated procedure used for the simulation of self-assembling (branched) polymers in solution^20^,^21^. In particular, the achiral (**1**), chiral (**2**) and reverse (2*) monomers were parameterized with the ‘general AMBER force field (GAFF)’ (*gaff.dat*)^40^. The atomistic models for the achiral (**1**), chiral (**2**) and reverse (2*) fibres were built starting from the 48 self-assembled prestacked cores previously optimized in vacuum by means of density functional theory calculations (BTA intercore distance of 3.4 Å)^24^. Three extended lateral chains, C_12_-PEG_4_-OH for the achiral (**1**) or *(R)-*3-Me-C_12_-PEG_4_-OH for the chiral (**2**) and *(S)-*3-Me-C_12_-PEG_4_-OH the reverse (2*) fibres, were added around the same starting core geometry to each BTA core, obtaining the starting initial extended configurations for the three fibres (Fig. 3a). In the reverse (2*) fibre, the positions of the hydrogen atom and of the methyl group connected to the third carbon atom of the BTA side chains were simply switched. The molecular models for the fibres were immersed in a periodic simulation box containing explicit TIP3P water molecules^41^. The simulation box was initially designed extending 12 Å from the tips of the extended side chains of the BTAs on the XY plane, and grazing the terminal BTA cores in the direction of the *z* axis (main axis of the fibre). In this way, replicated in space, the molecular systems are representative of the ideal bulk sections of monodisperse fibres of infinite length (Fig. 3a).

After initial minimization, the three achiral (**1**), chiral (**2**) and reverse (2*) systems initially underwent 50 ps of MD simulation in NVT conditions (constant N: number of atoms, V: volume and T: temperature in the system) to reach the experimental temperature of 20 °C (293 K). During this step, the solute was maintained as fixed. As a next step, the restraint was removed from the lateral chains of the BTA, which were pre-equilibrated for 2 ns of MD simulation in NPT conditions (constant N: number of atoms, P: pressure and T: temperature in the system) at room temperature (*T*=20 °C) and 1 atm of pressure. After this phase, all restraints were removed and the systems underwent additional 400 ns of MD simulation in NPT conditions at room temperature (*T*=20 °C) and 1 atm of pressure. Owing to the anisotropic (1D) nature of the simulated fibre models, anisotropic pressure scaling was adopted to allow the sides of the simulation box to change independently during the NPT MD runs. In this way, the pressure was kept constant inside the simulation box, and the fibre was free to rearrange and fold during the MD runs. All MD simulations used a time step of 2 fs, the Langevin thermostat and a 10-Å cutoff. The particle mesh Ewald^42^ approach was used to treat the long-range electrostatic effects, and all bonds involving Hydrogen atoms were treated with the SHAKE algorithm^43^. The fibre length, the root mean square displacement, the solvent-accessible surface area (SASA), as well as the fibre energy data were used to assess the equilibration of the simulated systems in the MD regime (see SI). Data show that all systems reach the equilibrium with after ≈300 ns of MD simulation. The last 100 ns of MD simulations were thus considered as representative of the equilibrated BTA fibres and used for structural and energetic analysis. The structural and hydrogen bonding analyses were conducted with the *ptraj* module of AMBER 12.

MD simulations of the BTA monomers in water were also conducted. The initially extended monomers were immersed in a periodic box containing explicit water molecules, and each system was first minimized and then equilibrated as previously described for 500 ns of NPT MD simulations. During the MD runs, the monomers underwent folding in solution and these simulations provided equilibrated trajectories for the monomers dissolved in solution.

The energy analysis was performed according to the MM-GBSA approach^44^,^45^. In particular, the average molecule self-assembly energies Δ*E* were calculated from the MD trajectories of the simulated fibre systems and of the BTA monomers as:


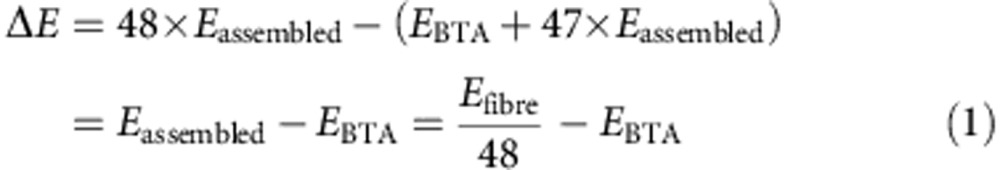


Where *E*_assembled_ is the average energy of the BTAs in the fibre assembly (which can be extracted from the energy of the fibre *E*_fibre_) and *E*_BTA_ is the energy of the BTA monomers as dissolved in solution. Thus, Δ*E* data compares the average energy of one state where 48 BTA molecules assembled in the fibre, with a state where 47 BTAs are assembled and one BTA is dissolved in solution. According to equation (1), this is consistent to compare the average energy of one BTA in the assembled fibre with that of the monomer dissolved in solution. Δ*E* is thus representative of the energy gain accompanying self-assembly or of the incorporation of one BTA from the solution in the self-assembled structure. As calculated Δ*E* values might depend on the number of BTAs in the system, the values reported in Figs 4 and 5 were normalized per-BTA molecule in the simulated system (average Δ*E* red dotted lines). The extracted Δ*E* values account for solute–solute and solute–solvent interactions, and are calculated as:


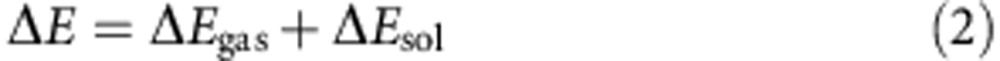


Where Δ*E*_gas_ is the total gas-phase (in vacuum) non-bond energy and Δ*E*_sol_ is the solvation term calculated as: Δ*E*_sol_=Δ*E*_GB_+Δ*E*_NP_^46^. The polar component of solvation (Δ*E*_GB_) was evaluated according to the generalized Born approach^47^,^48^. The non-polar contribution to the solvation energy was calculated as Δ*E*_NP_=*g* (SASA)+*b*, where *g*=0.00542, kcal Å^−2^, *b*=0.92 kcal mol^−1^ and SASA is the solvent-accessible surface that was estimated with the MSMS programme^49^.

The Δ*E*_*n*_ energy contributions to self-assembly of the individual 48 BTA molecules in the fibre models (Figs 4 and 5) were calculated from the total self-assembly energy of the simulated fibre according to per-residue decomposition^50^,^51^. This allowed us to obtain insight on the order and the relative stability of the different BTAs in the fibre (Figs 4 and 5). Moreover, the total Δ*E* energies were also decomposed on a pairwise residue basis, allowing one to study the pair BTA–BTA interactions between each monomer and the other ones in the fibre on an individual level (Supplementary Figs 31 and 32). This provided insight on the average interaction energies of the BTAs with their closest neighbours in the assembled fibres, which are those directly involved in the BTA–BTA stacking and hydrogen bonding. In general, the pair BTA–BTA interaction Δ*E*_int_ is stronger for the closest neighbours, and rapidly decreases and drifts to zero for farther BTAs in the fibre. Distribution plots of the BTAs Δ*E*_int_ as a function of the position of the neighbours (of their distance in the self-assembled scheme) are reported in Supplementary Fig. 32.

We also created one additional molecular system composed of seven monodisperse BTA molecules prefolded in solution (final configurations of the monomer simulations) immersed in a simulation box containing explicit water molecules. After preliminary minimization, this system underwent 400 ns of MD simulation in NPT condition. During the simulation, the BTAs undergo aggregation, and after ≈150 ns of simulation the cores of three BTAs form a stable stacking with complete and persistent hydrogen bonding motif (see SI). This simulation was conducted for the achiral (**1**) monomers, and used to assess that our set-up was able to capture the spontaneous self-assembly of dissolved BTAs in solution; further analysis of these results will be presented at a later time.

## Author contributions

M.B.B, L.A., G.M.P., A.R.A.P. and E.W.M. conceived the project and contributed to the concept of the manuscript. M.B.B, L.A., I.K.V. and G.M.P. designed the experiments and analysed the results; computational work was performed by G.M.P. C.M.A.L. provided compound **2** and contributed to discussions on the project. M.B.B, L.A. and G.M.P. wrote the manuscript and managed the project.

## Additional information

**How to cite this article**: Baker, M. B. *et al.* Consequences of chirality on the dynamics of a water-soluble supramolecular polymer. *Nat. Commun.* 6:6234 doi: 10.1038/ncomms7234 (2015).

## Supplementary Material

Supplementary Figures, Supplementary Methods and Supplementary References Supplementary Figures 1-35, Supplementary Methods and Supplementary References

Supplementary Movie 1 Detail of the relaxation MD simulation of the achiral (1) fiber in water solution at 20 °C. The fiber model is replicated infinitely along the Z axis according to the periodic boundary conditions (only the BTA cores are shown for the replicated system). Starting from an ideal linear displacement of open BTAs, the fiber (1) rapidly starts to fold in water (cyan).

Supplementary Movie 2 Detail of the relaxation MD simulation of the chiral (2) fiber in water solution at 20 °C. The fiber model is replicated infinitely along the Z axis according to the periodic boundary conditions. The methyl groups (red) surround the BTA cores (black). Starting from an ideal linear displacement of open BTAs, fiber (1) rapidly start to fold in water (cyan).

Supplementary Movie 3 Relaxation MD simulation of the chiral (2) fiber in water at 20 °C. The (2) BTA cores are colored in black and the methyl groups in red. The side chains are colored in green in the simulated system, and in transparent grey in the replicated images. The clearly visible black patches in the simulated system (green side chains) identify regions where the fiber core is accessible by water molecules from the external solution.

Supplementary Movie 4 Spontaneous self-assembly of seven pre-folded BTA in solution. During 400 ns of MD simulation, the seven BTAs undergo aggregation. After ~150 ns, three BTAs form a stable stacked trimer with formation of stable Hydrogen bonds (pink)

## Figures and Tables

**Table 1:** Structural information from SAXS*.

**Fibre**	**Kuhn length (nm)**	**Length (nm)**	**Radius (nm)**	**Dispersity (nm)**	***χ***^**2**^**/*****N***
**(1)**	19.1±17.3	191.4±2.9	3.1±0.0	0.01±0.01	2,118
**(2)**	63.6±61.1	191.4±439.6	3.1±0.0	0.01±0.01	4,004

SAXS, small-angle X-ray scattering.

^*^achiral (**1**) measured as 0.45 wt% in H_2_O and chiral (**2**) measured as 0.25 wt% in 96% H_2_O/4% MeOH. Values are obtained from fitting with the Schurtenberger–Pedersen form factor.

**Table 2:** Structural information from MD simulation.

**Fibre**	**Intercore BTA distance (Å)**	**Average dihedral angle**	**Equilibrated length** **(nm)**	Average thickness* **(nm)**	**Persistent H-bonds**†	**% Of H-bonds in initial helicity**‡
**(1)**	≈3.7–3.8	−66.0°	7.1	3.6–4.7	39	45
**(2)**	≈3.4–3.5	−98.8°	9.5	3.5–3.8	56	76
(2*)	≈3.6–3.7	−85.2°	7.2	3.8–5.0	48	58

MD, molecular dynamics.

^*^Average thickness may vary depending on the sections of the fibres.

^†^Number of hydrogen bonds present for more than 95% of time during the last 100 ns of the equilibrated MD simulations.

^‡^Percentage of H-bonds remaining in the initial starting *P-*helicity; (−90° to −180°).

**Table 3:** Energetic information from MD simulation.

**Fibre**	**Δ*****E***_int_* **(kcal mol**^−**1**^**)**	**Δ*****E***_**int,sol**_† **(kcal mol**^−**1**^**)**	**Δ*****E***_**int,sol**_**/Δ*****E***_**ele**_	**HBs/BTA**‡	**Δ*****E***_**HBs**_§ **(kcal mol**^−**1**^**)**	**Δ*****E***_**sol**_|| **(kcal mol**^−**1**^**)**	**Δ*****E***_**sol**_**/Δ*****E***_**HBs**_
**(1)**	−38.1±5.8	−10.8±2.2	2.1	1.6	−2.6	−39.5	15.2
**(2)**	−34.8±5.2	−10.0±1.7	2.0	2.3	−3.7	−38.9	10.5
(2*)	−33.4±6.2	−9.6±2.3	2.0	2.0	−3.2	−40.3	12.6

^*^Average pairwise interaction energy of the BTAs with the closest neighbours in the self-assembled structure.

^†^Solvation term of Δ***E***_**int**_(accounting for closest neighbours only).

^‡^Number of persistent H-bonds per-BTA (Table 1) multiplied per two (each BTA interacts with two neighbours in the stacking).

^§^Number of persistent stacking H-bonds per-BTA multiplied for the typical energy value of a peptide H-bond in water (−1.58 kcal mol^−1^)^37^.

^||^Solvation energy accounting for all BTAs in the fibre.
