# Supplementary material for: Consequences of chirality on the dynamics of a water-soluble supramolecular polymer
Source: Nat Commun. 2015 Feb 20;6:6234. doi: 10.1038/ncomms7234 (PMC4346625; doi:10.1038/ncomms7234)
Supplement: Supplementary Figures, Supplementary Methods and Supplementary References — Supplementary Figures 1-35, Supplementary Methods and Supplementary References [file ncomms7234-s1.pdf]

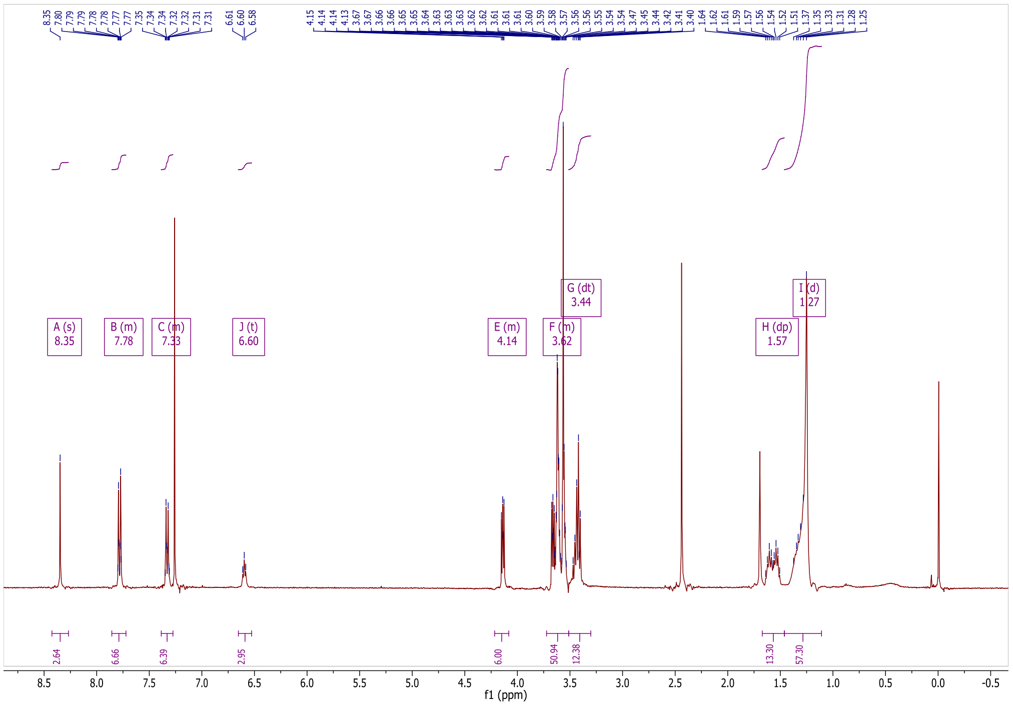

Supplementary Figure 1: ^{1}H NMR of S1 in $CDCl_3$

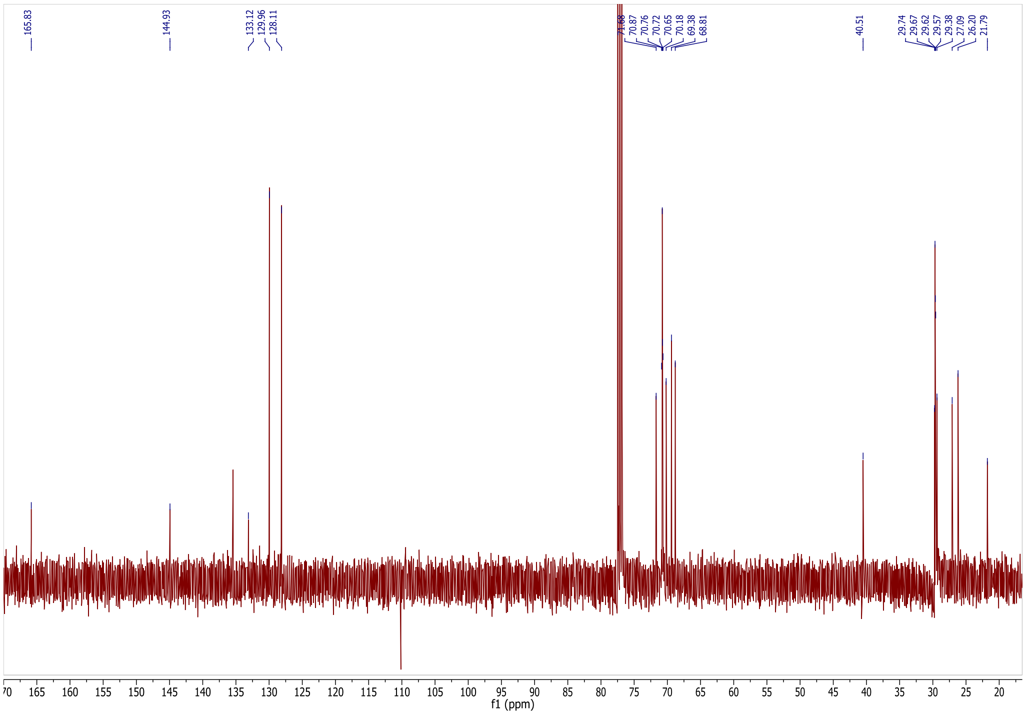

Supplementary Figure 2: ^{13}C NMR of S1 in $CDCl_3$

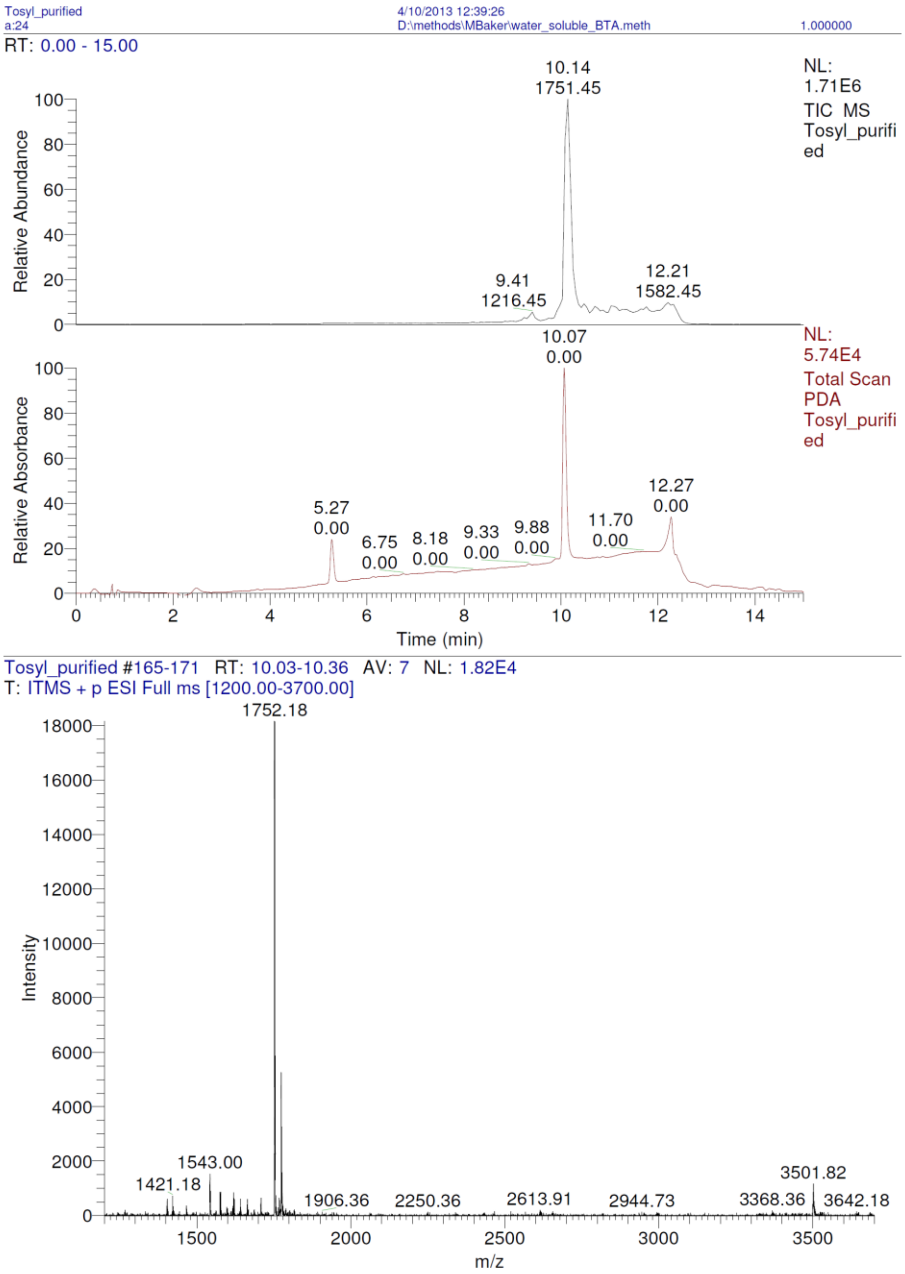

Supplementary Figure 3: LC/MS of S1

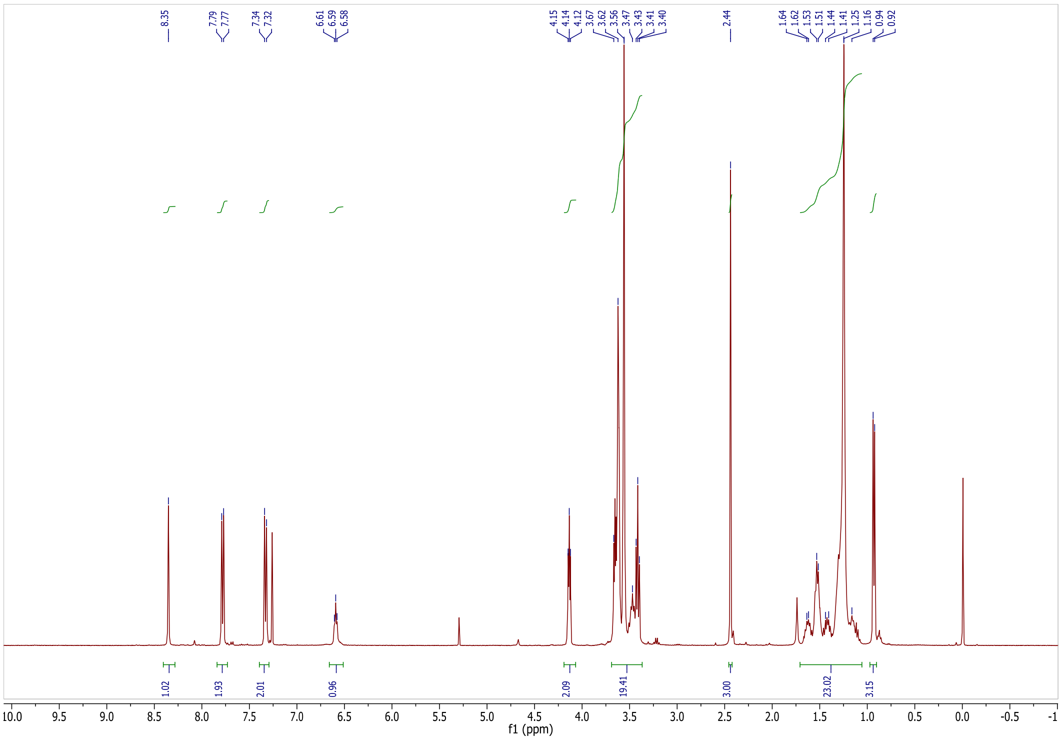

Supplementary Figure 4: ^{1}H NMR of S2 in $CDCl_3$

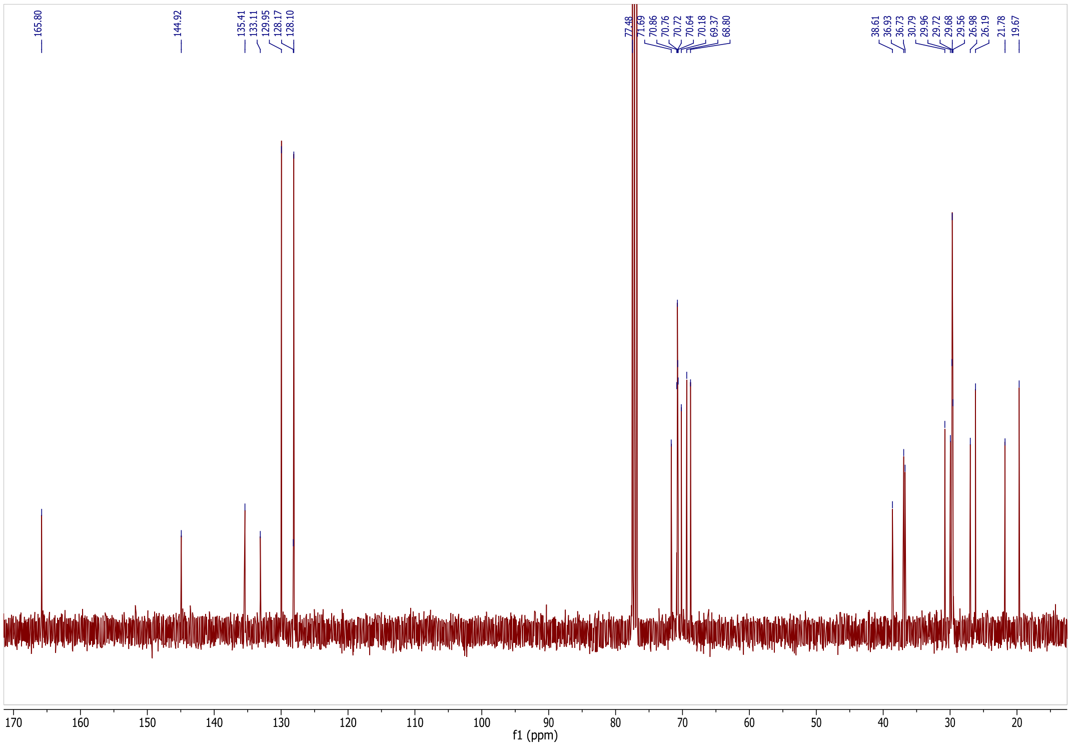

Supplementary Figure 5: ^{13}C NMR of S2 in $CDCl_3$

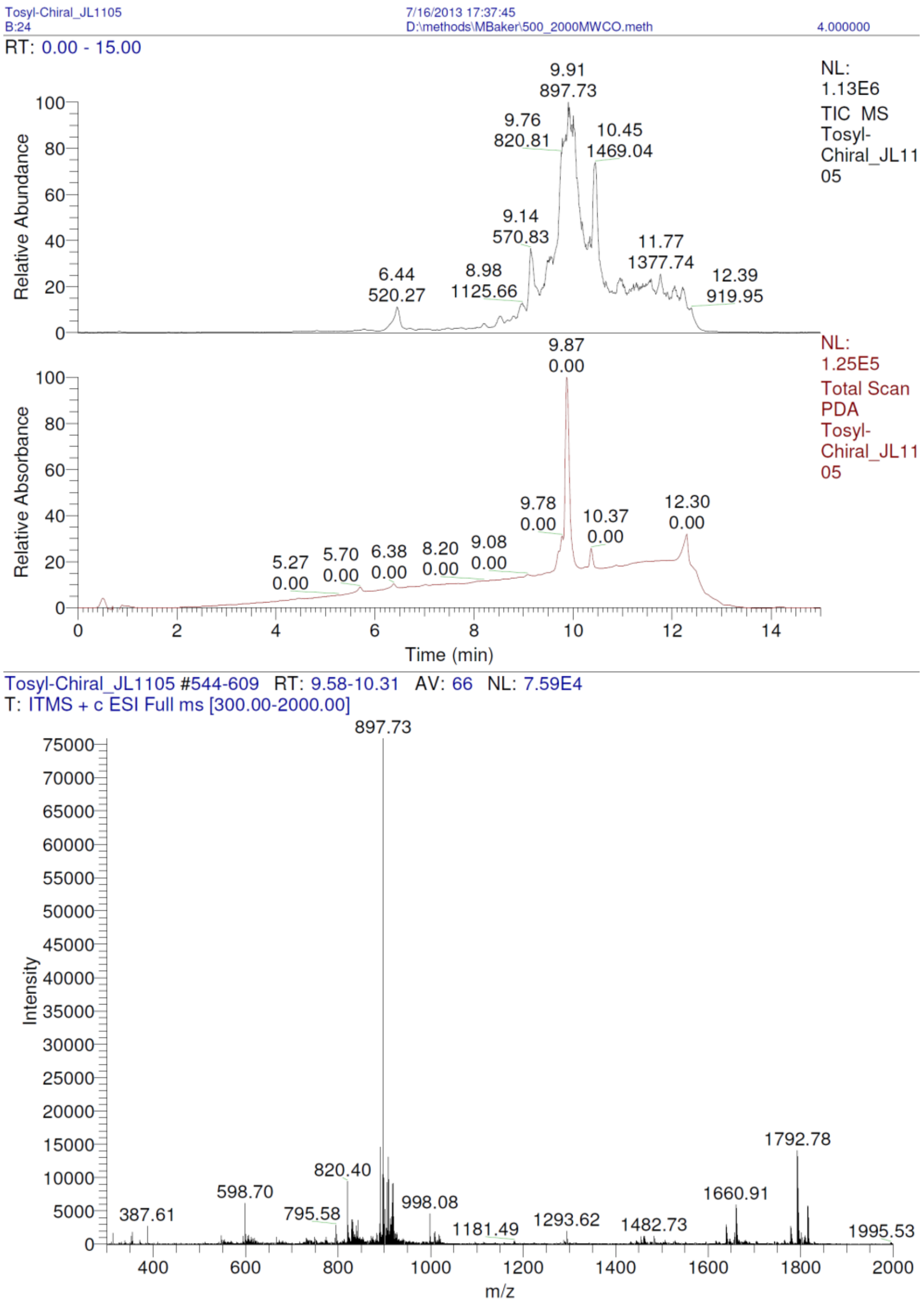

Supplementary Figure 6: LC/MS of S2

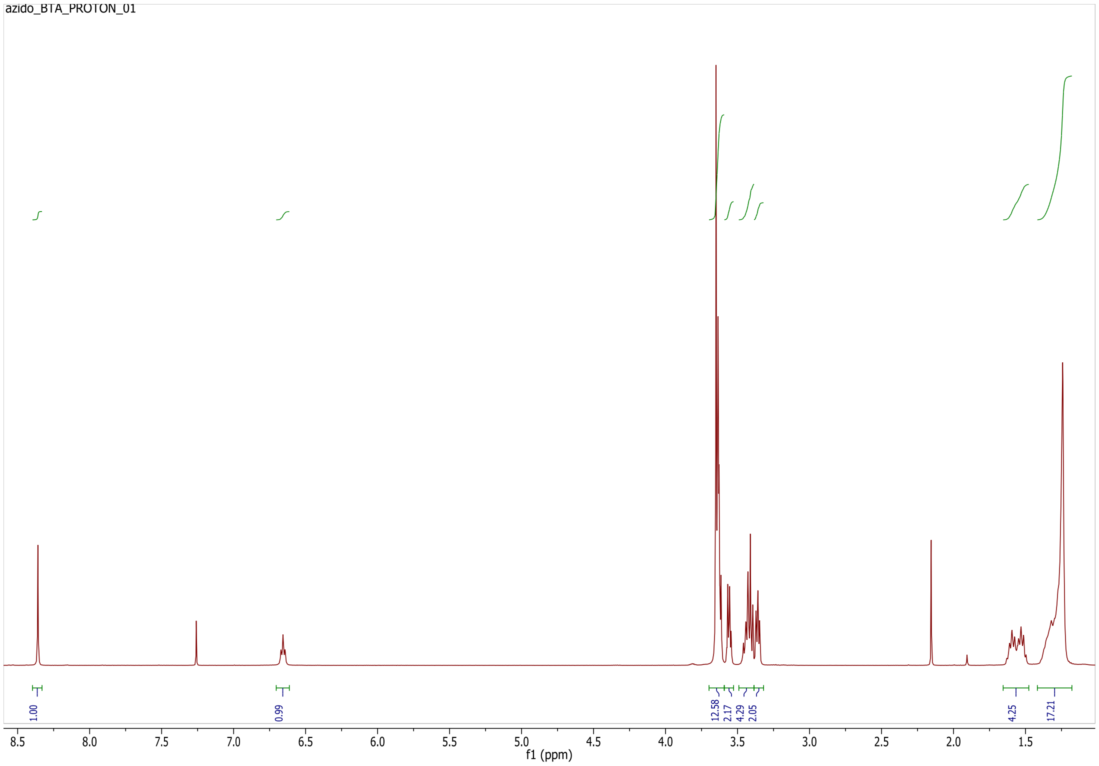

Supplementary Figure 7: ^{1}H NMR of S3 in $CDCl_3$

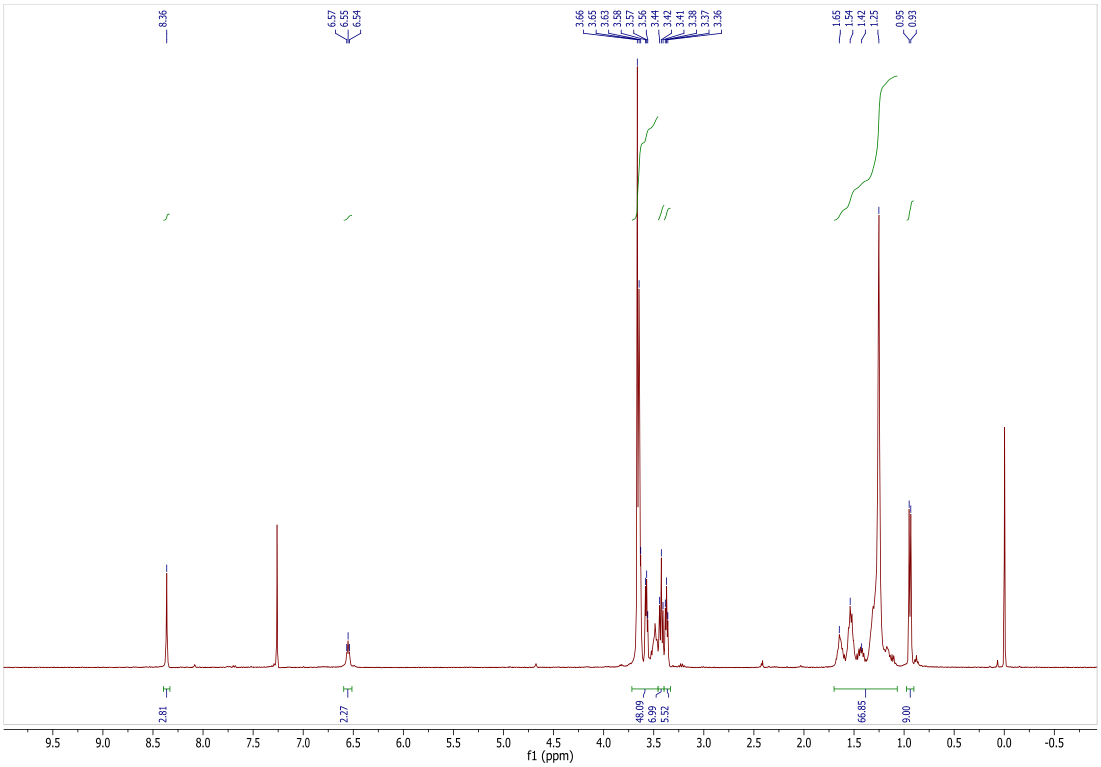

Supplementary Figure 8: ^{1}H NMR of S4 in $CDCl_3$

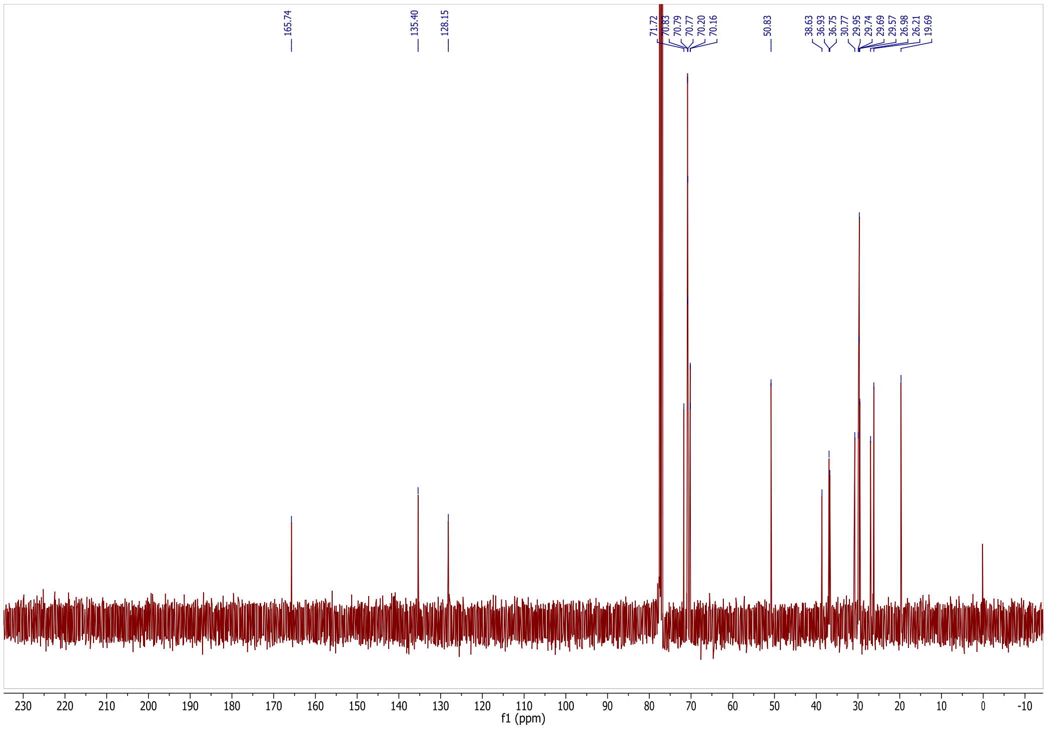

Supplementary Figure 9: ^{13}C NMR of S4 in $CDCl_3$

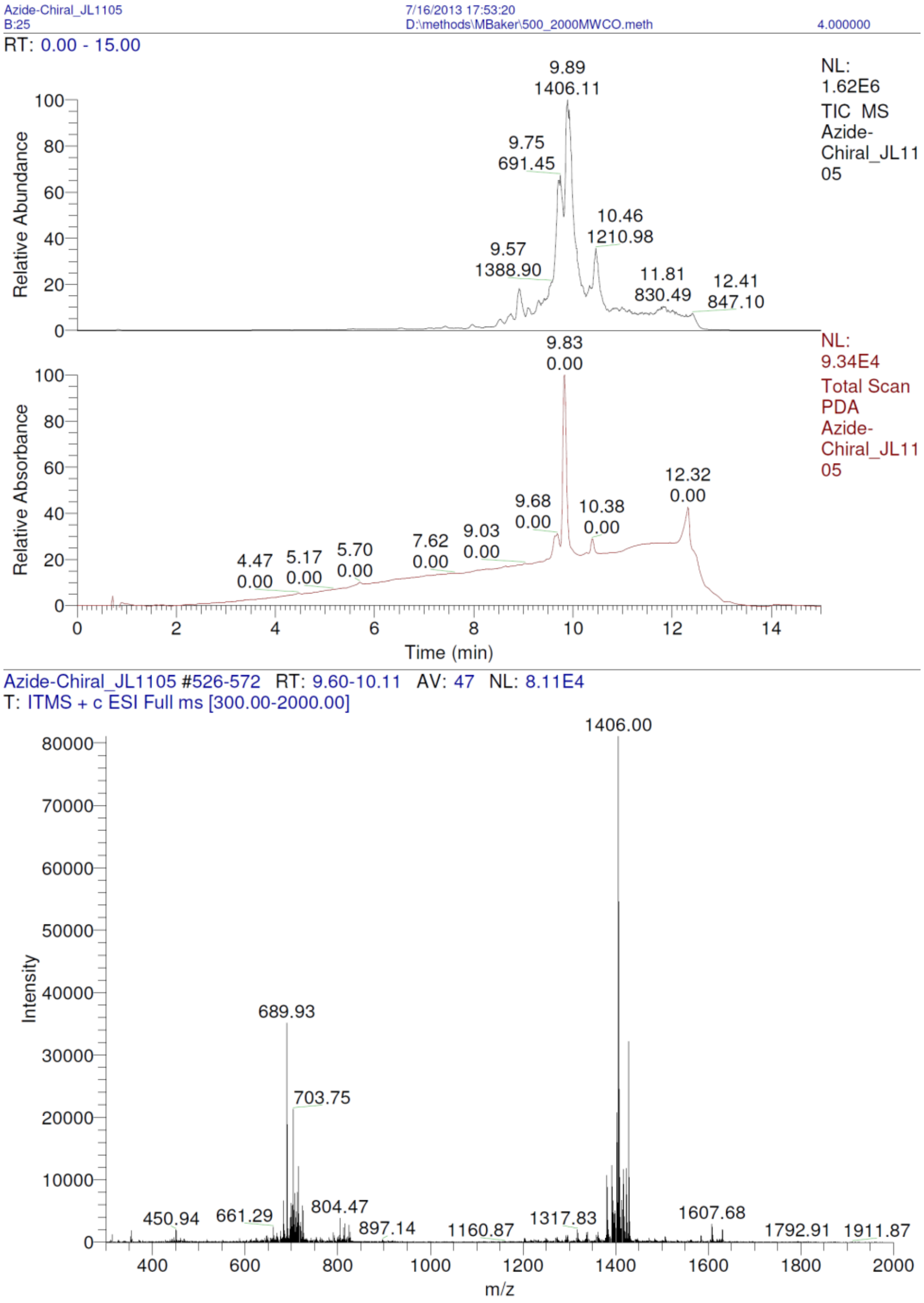

Supplementary Figure 10: LC/MS of S4

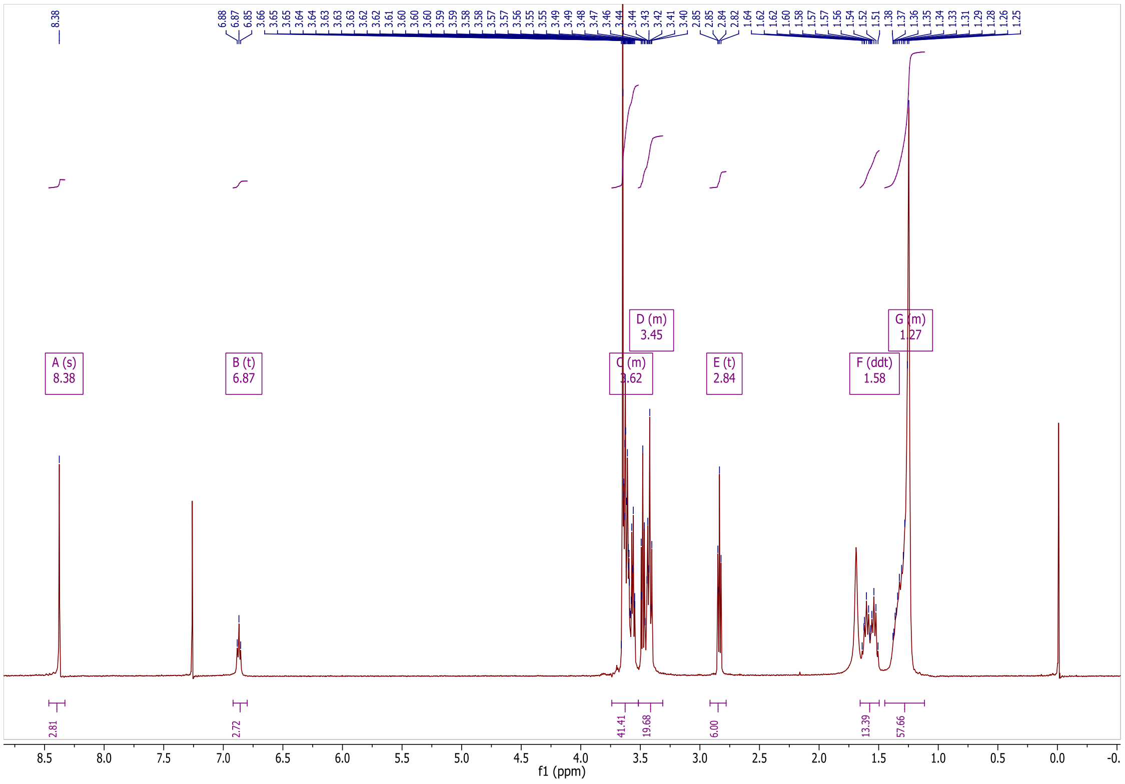

Supplementary Figure 11: ^{1}H NMR of S5 in $CDCl_3$

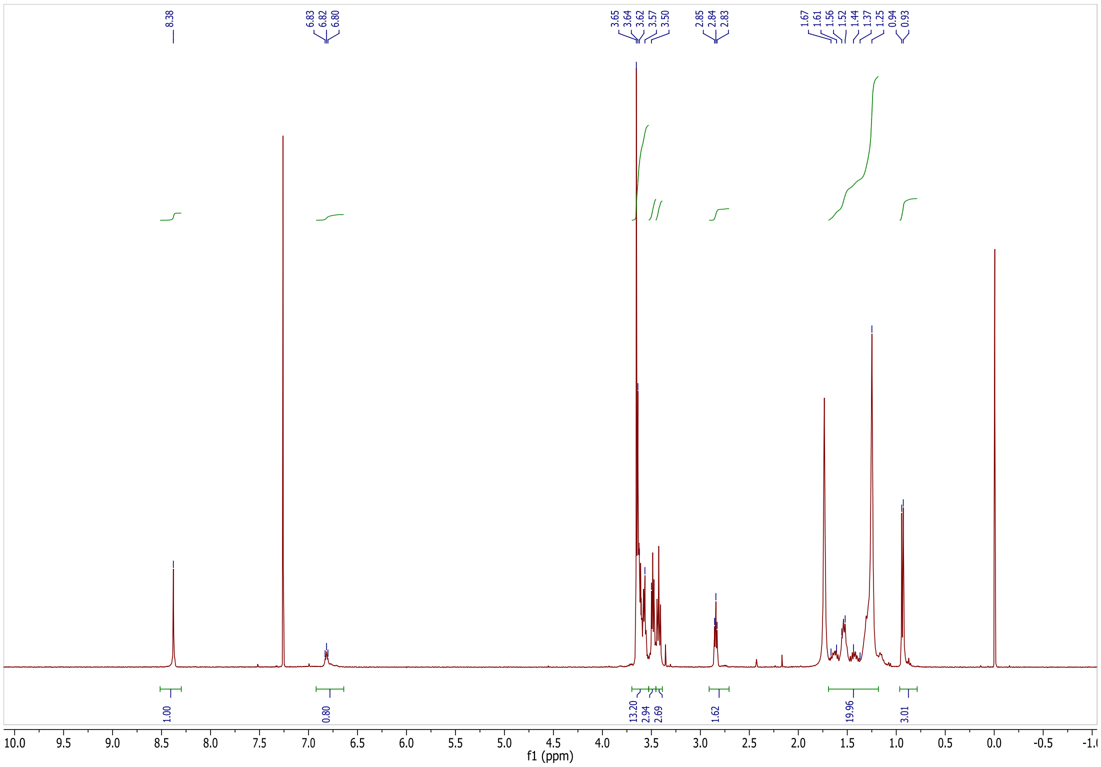

Supplementary Figure 12: ^{1}H NMR of S6 in $CDCl_3$

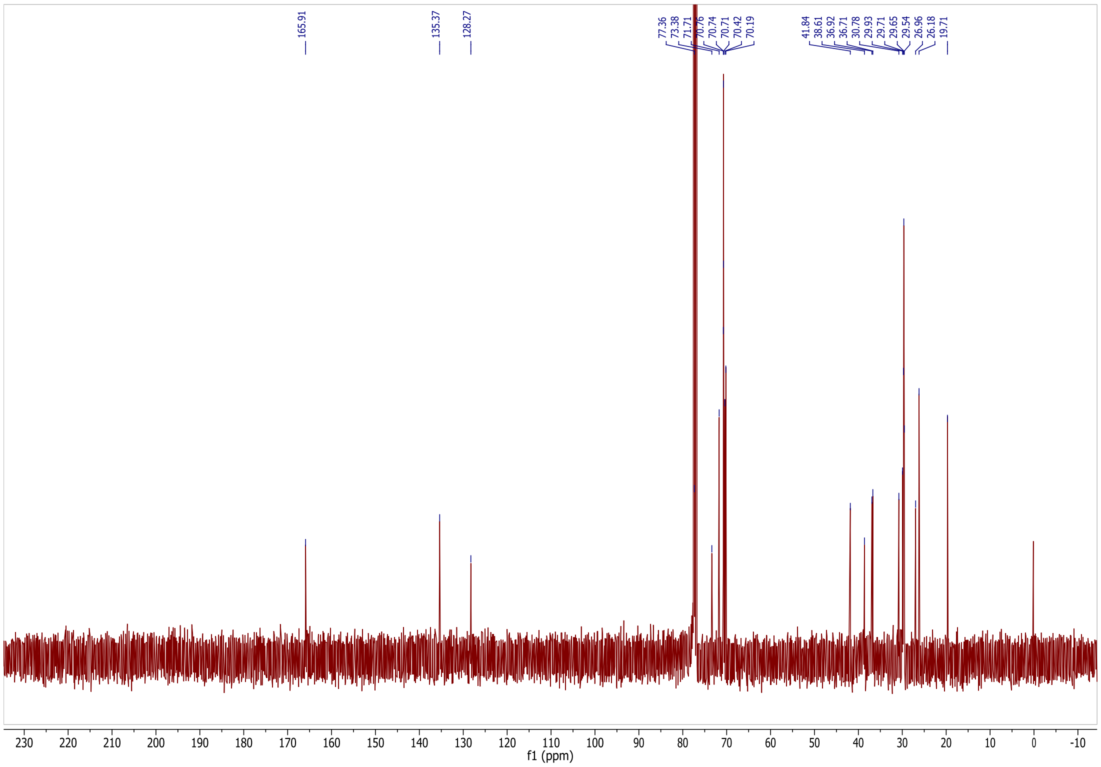

Supplementary Figure 13: ^{13}C NMR of S6 in $CDCl_3$

D:\Data\Users 2013\Ralf\September\Matt_B\September042013\MBchiralNH2_CHCA_spotC3_laser_int40_detg7\0_C3\1\1Ref

Comment 1 CHCA/DCTB in THF calibration in D:\Data\Users 2013\Ralf\September\Matt_B\September042013

Comment 2

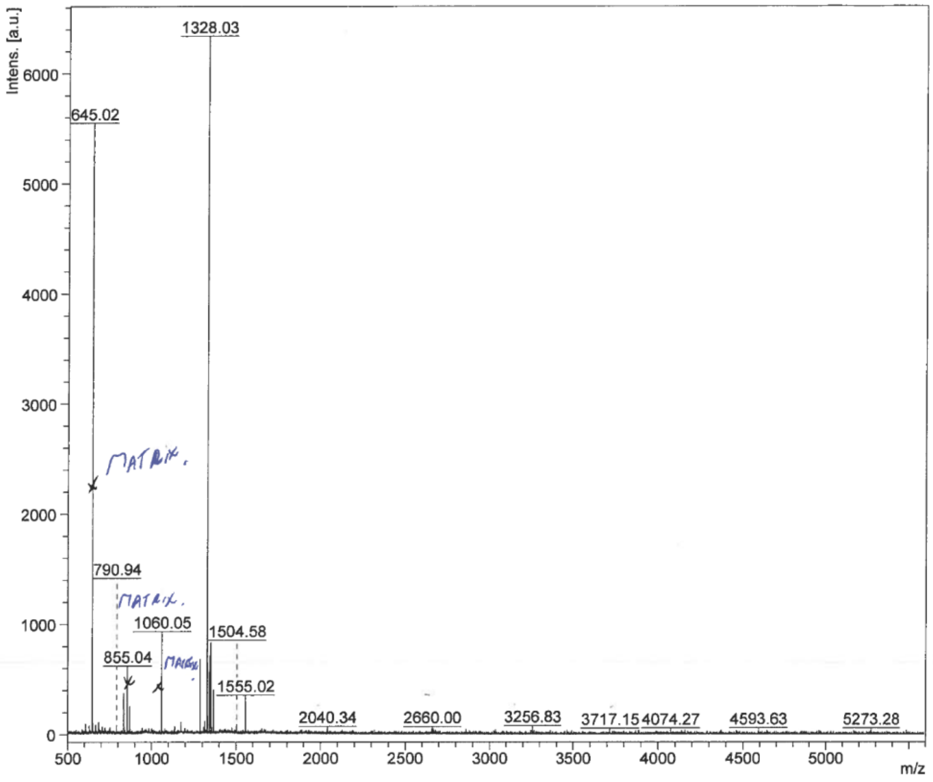

Acquisition Parameter

Date of acquisition	2013-09-04T15:05:19.729+01:00
Acquisition method name	D:\Methods_old\flexControlMethods\RP_700-3500_Da.par
Aquisition operation mode	Reflector
Voltage polarity	POS
Number of shots	500
Name of spectrum used for calibration	
Calibration reference list used	Cesium tri jodide positive mode

Instrument Info

User	smo379
Instrument	ST-A2130
Instrument type	autoflex

Bruker Daltonics flexAnalysis printed: 04-Sep-13 15:11:39

Supplementary Figure 14: MALDI of S6

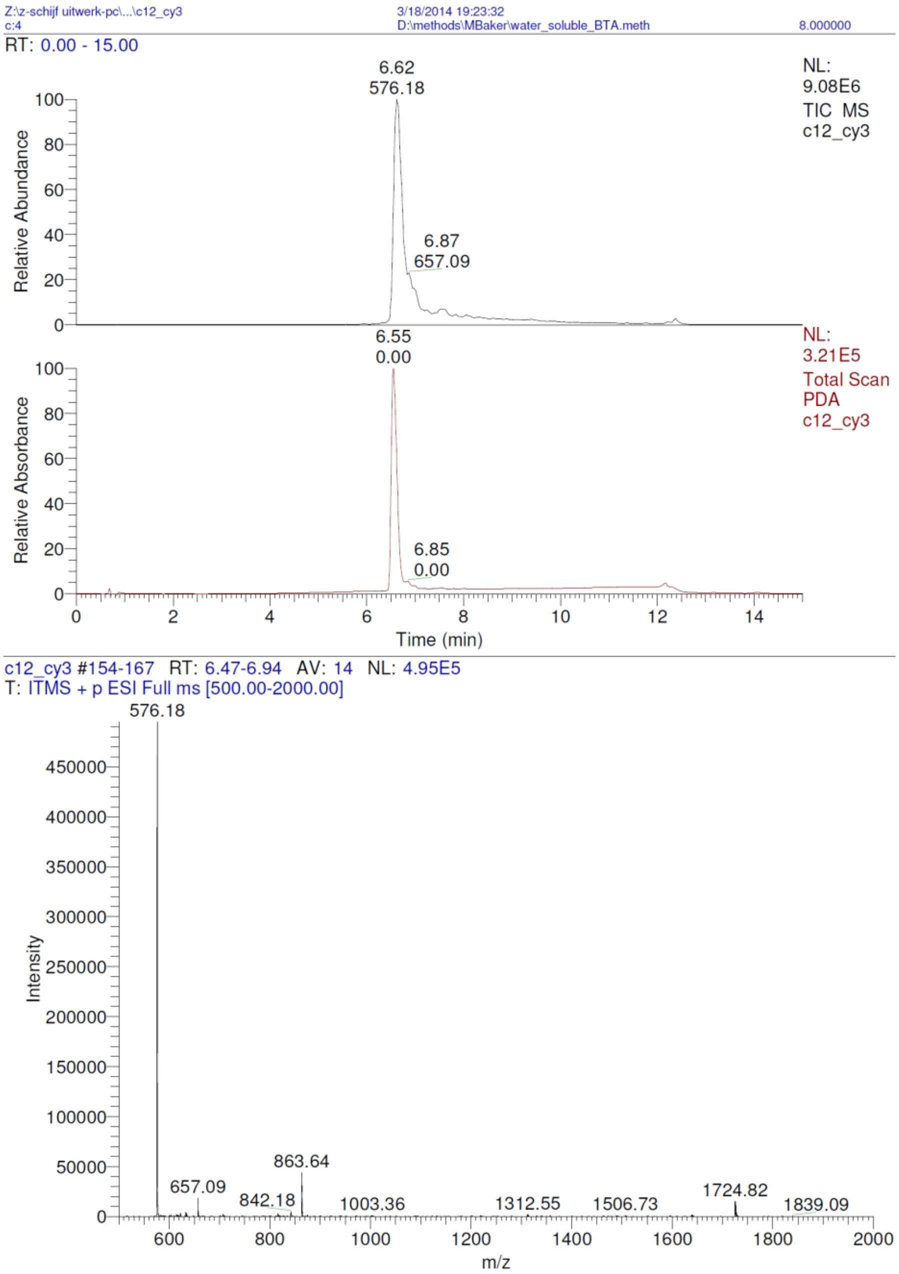

Supplementary Figure 15: LC/MS of 3a

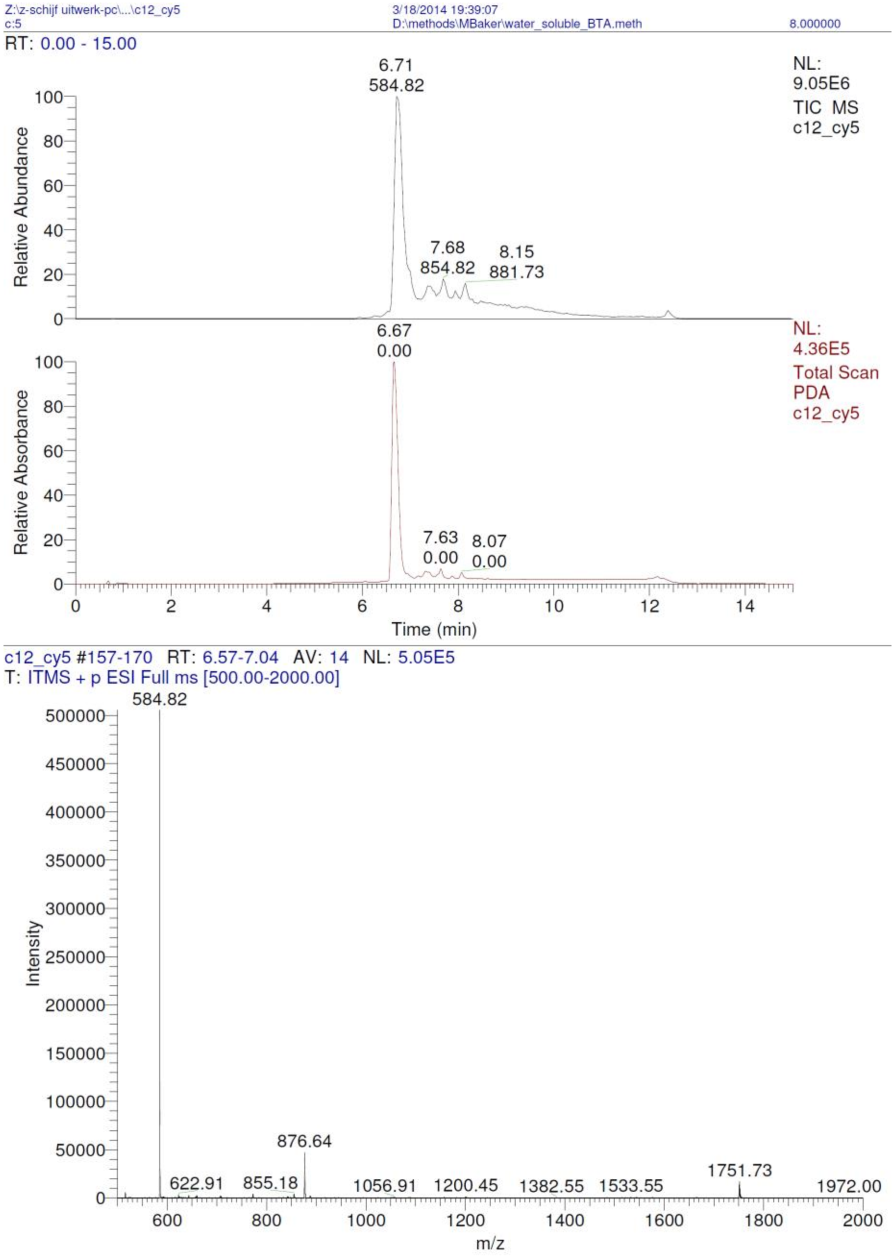

Supplementary Figure 16: LC/MS of 3b

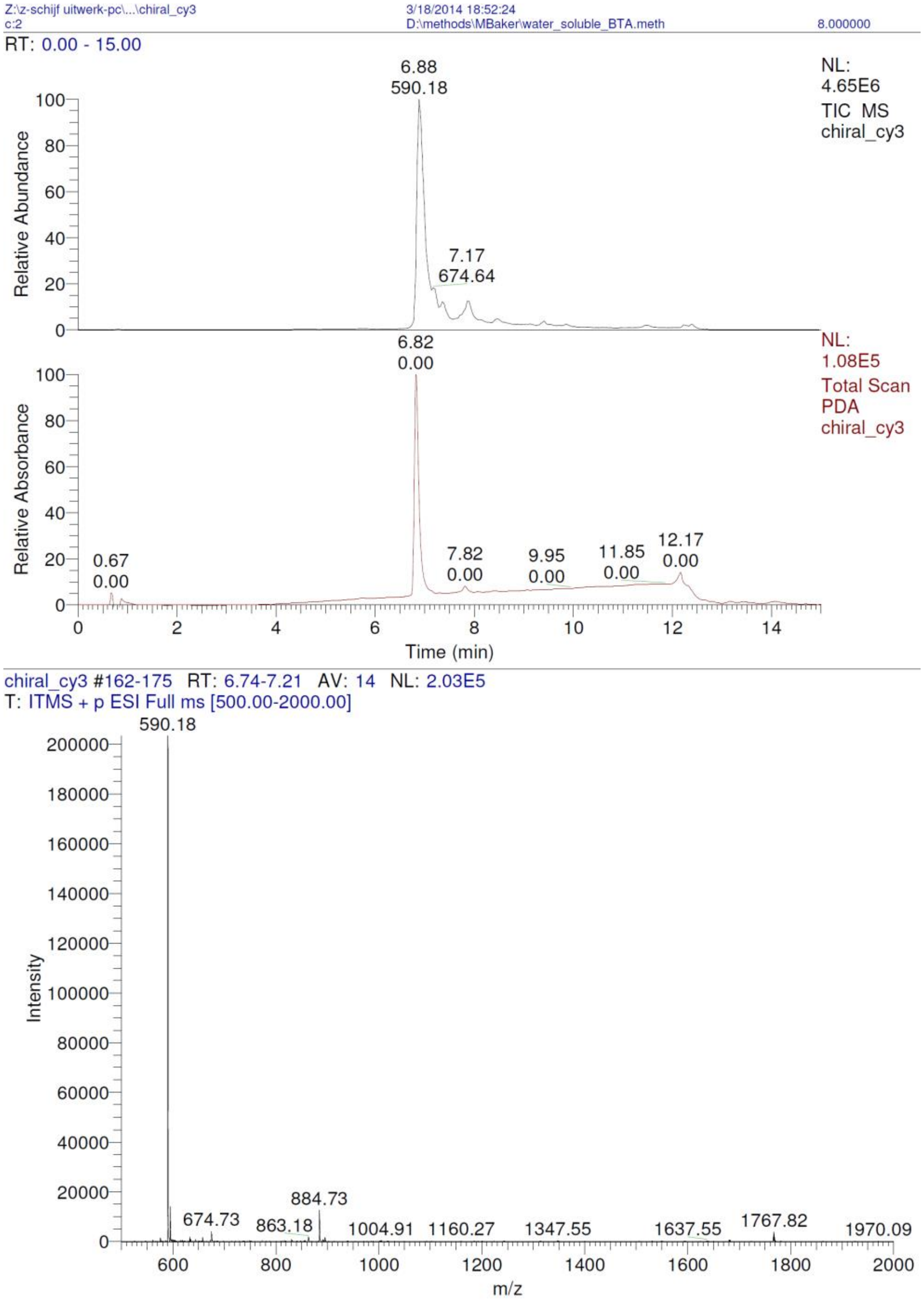

Supplementary Figure 17: LC/MS of 4a

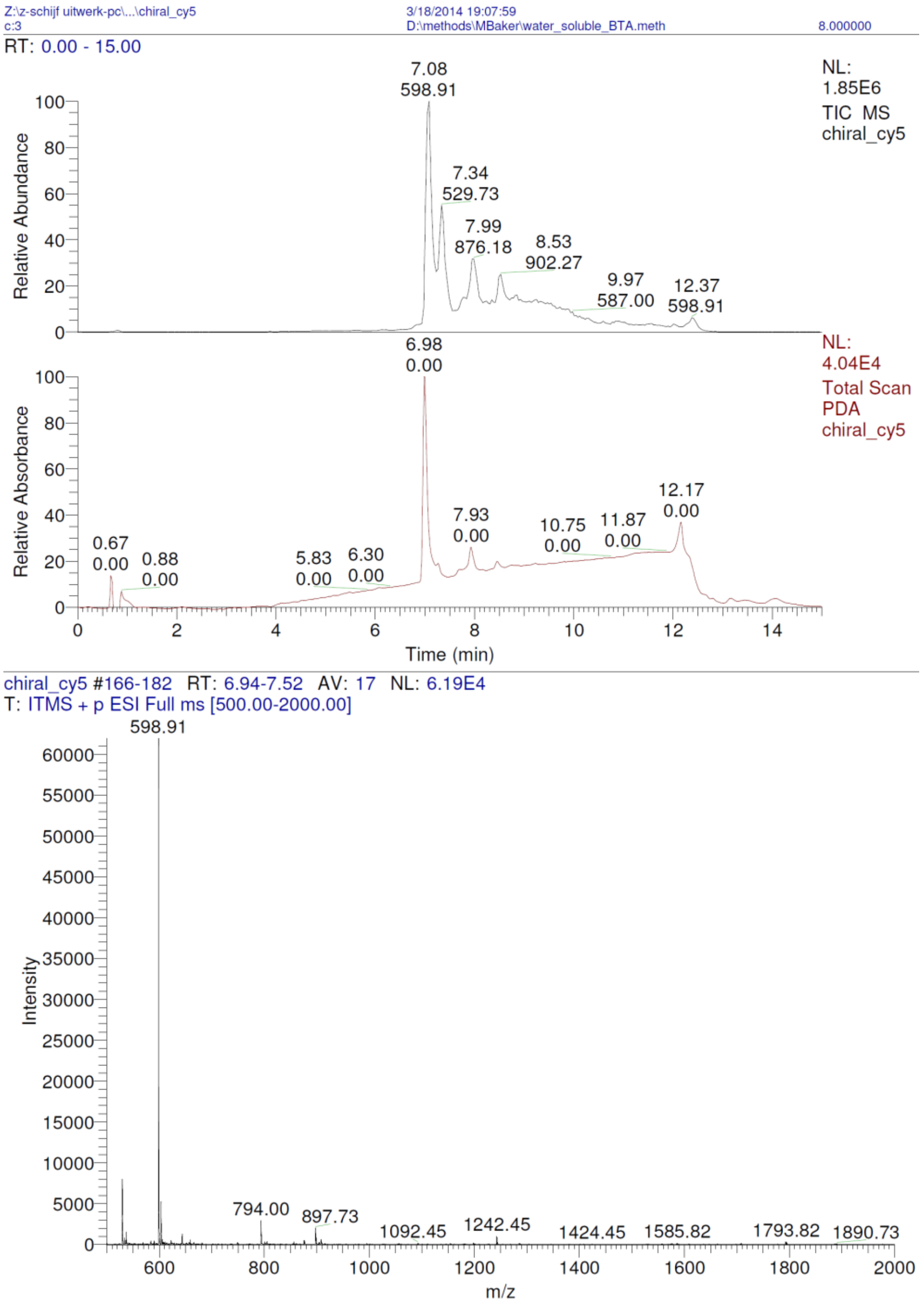

Supplementary Figure 18: LC/MS of 4b

FRET Mixing studies

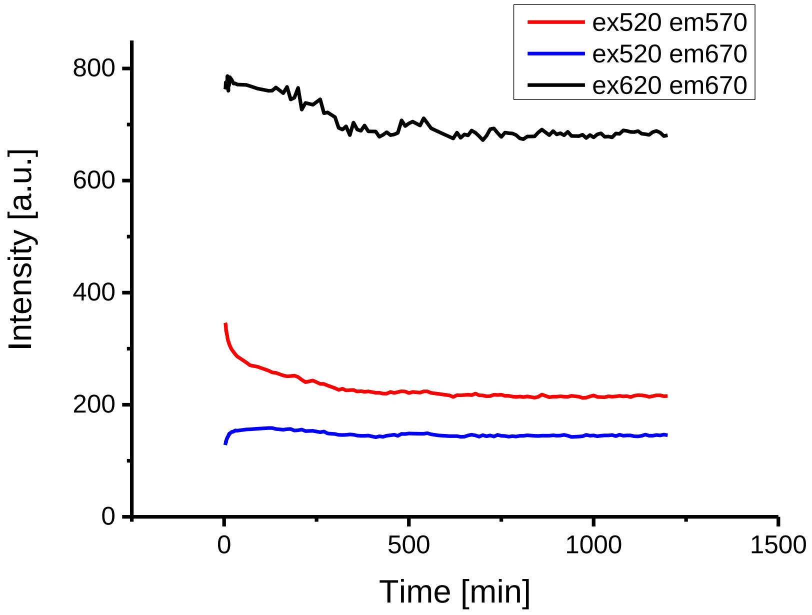

Supplementary Figure 19: Representative raw data from FRET exchange experiment of achiral (1). Red curve is excitation at 520 nm and emission at 570 (Cy3 excitation and emission); blue curve is excitation at 520 nm and emission at 670 nm (Cy3 excitation and Cy5 emission); black curve is excitation at 620 nm and emission at 670 nm (Cy5 excitation and emission).

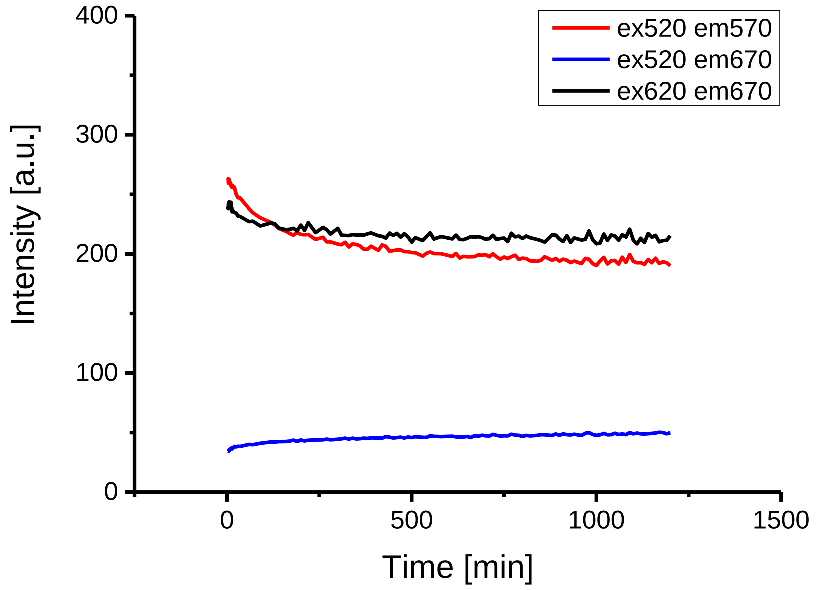

Supplementary Figure 20: Representative raw data from FRET exchange experiment of chiral (2). Red curve is excitation at 520 nm and emission at 570 (Cy3 excitation and emission); blue curve is excitation at 520 nm and emission at 670 nm (Cy3 excitation and Cy5 emission); black curve is excitation at 620 nm and emission at 670 nm (Cy5 excitation and emission).

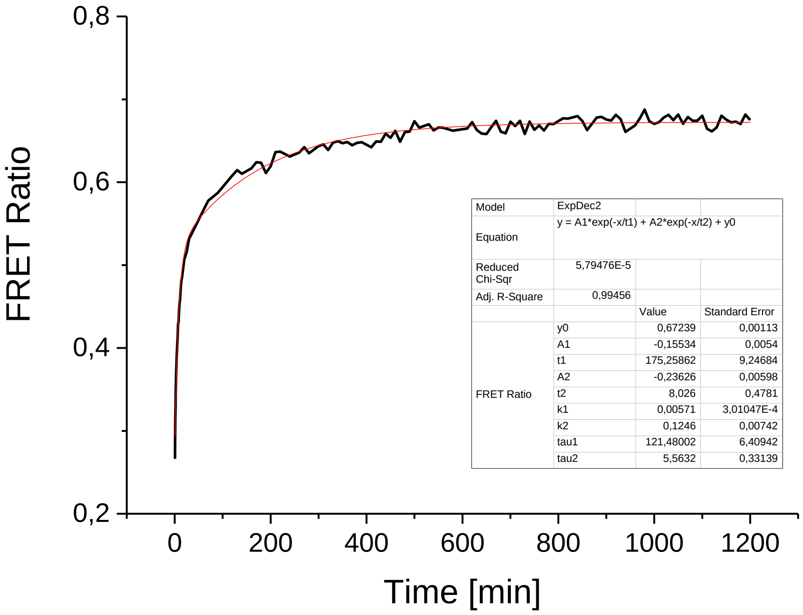

Supplementary Figure 21: Curve fitting of FRET exchange for 1

Model ExpDec2			
Equation		y = A1*exp(-x/t1) + A2*exp(-x/t2) + y0	
Reduced Chi-Sqr		5,79476E-5	
Adj. R-Square		0,99456	
		Value	Standard Error
FRET Ratio	y0	0,67239	0,00113
FRET Ratio	A1	-0,15534	0,0054
FRET Ratio	t1	175,25862	9,24684
FRET Ratio	A2	-0,23626	0,00598
FRET Ratio	t2	8,026	0,4781
FRET Ratio	k1	0,00571	3,01047E-4
FRET Ratio	k2	0,1246	0,00742
FRET Ratio	tau1	121,48002	6,40942
FRET Ratio	tau2	5,5632	0,33139

Supplementary Table 1: Fitting parameters for exchange of 1

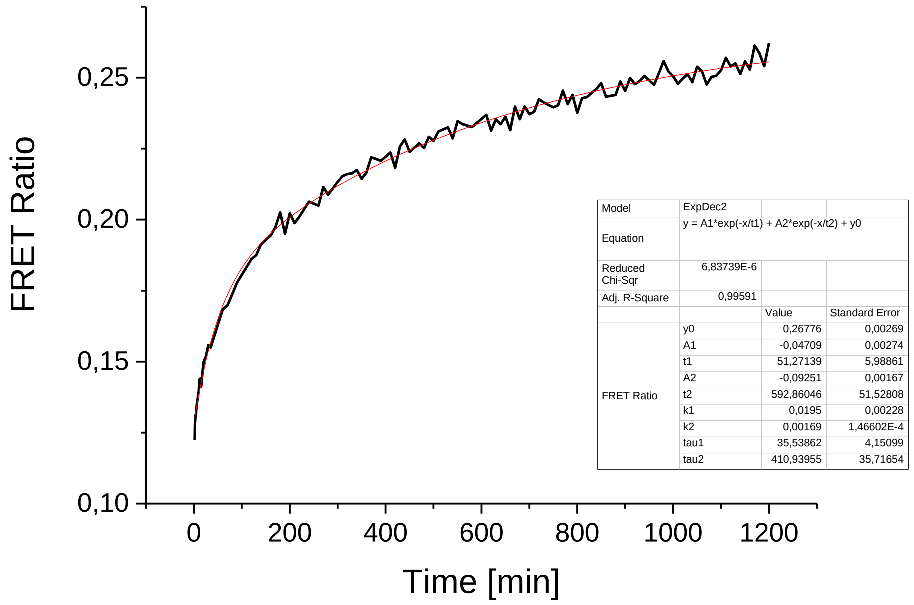

Supplementary Figure 22: Curve fitting of FRET exchange for 2

Model	ExpDec2		
Equation	y = A1*exp(-x/t1) + A2*exp(-x/t2) + y0		
Reduced Chi-Sqr		6,83739E-6	
Adj. R-Square		0,99591	
		Value	Standard Error
FRET Ratio	y0	0,26776	0,00269
FRET Ratio	A1	-0,04709	0,00274
FRET Ratio	t1	51,27139	5,98861
FRET Ratio	A2	-0,09251	0,00167
FRET Ratio	t2	592,86046	51,52808
FRET Ratio	k1	0,0195	0,00228
FRET Ratio	k2	0,00169	1,46602E-4
FRET Ratio	tau1	35,53862	4,15099
FRET Ratio	tau2	410,93955	35,71654

Supplementary Table 2: Fitting parameters for curve of 2

Molecular Dynamics Simulations

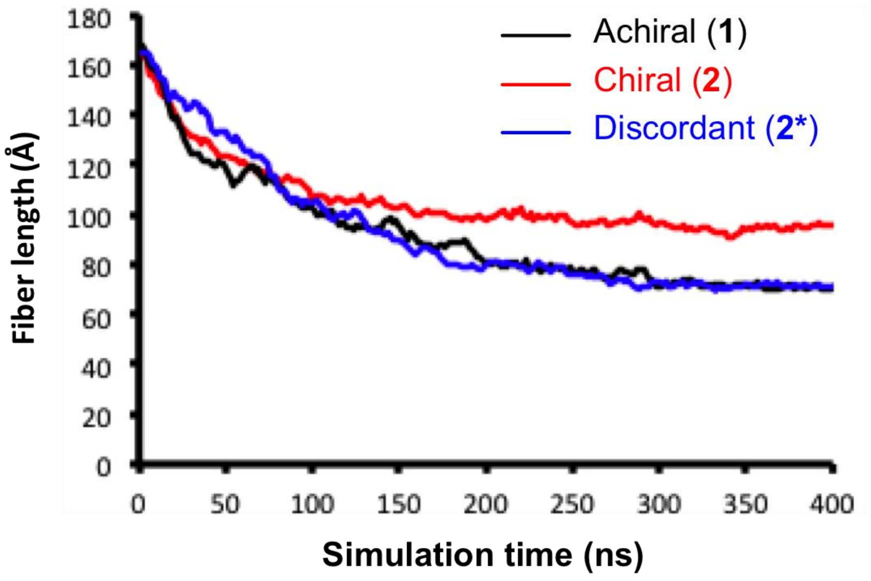

Supplementary Figure 23: Fiber length calculated from the MD simulations at variance of the simulation time

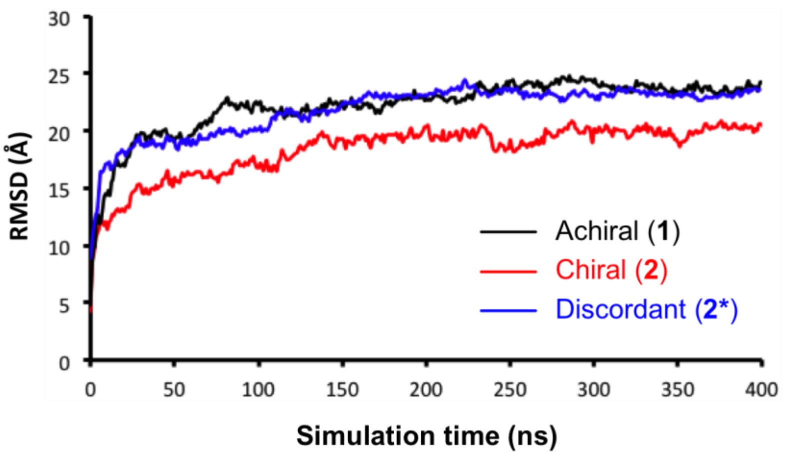

Supplementary Figure 24: RMSD data calculated from the MD simulations at variance of the simulation time

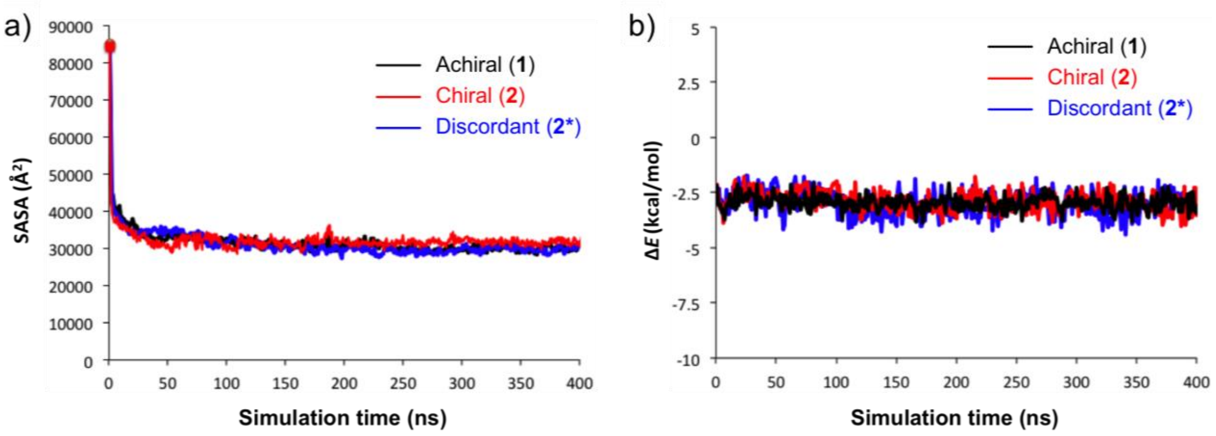

Supplementary Figure 25: Other data assessing the systems equilibration. (a) The solvent accessible surface area (SASA) of the fibers calculated as a function of the simulation time. (b) The average per-BTA self-assembling energy (Δ*E*) for all systems calculated as a function of the simulation time.

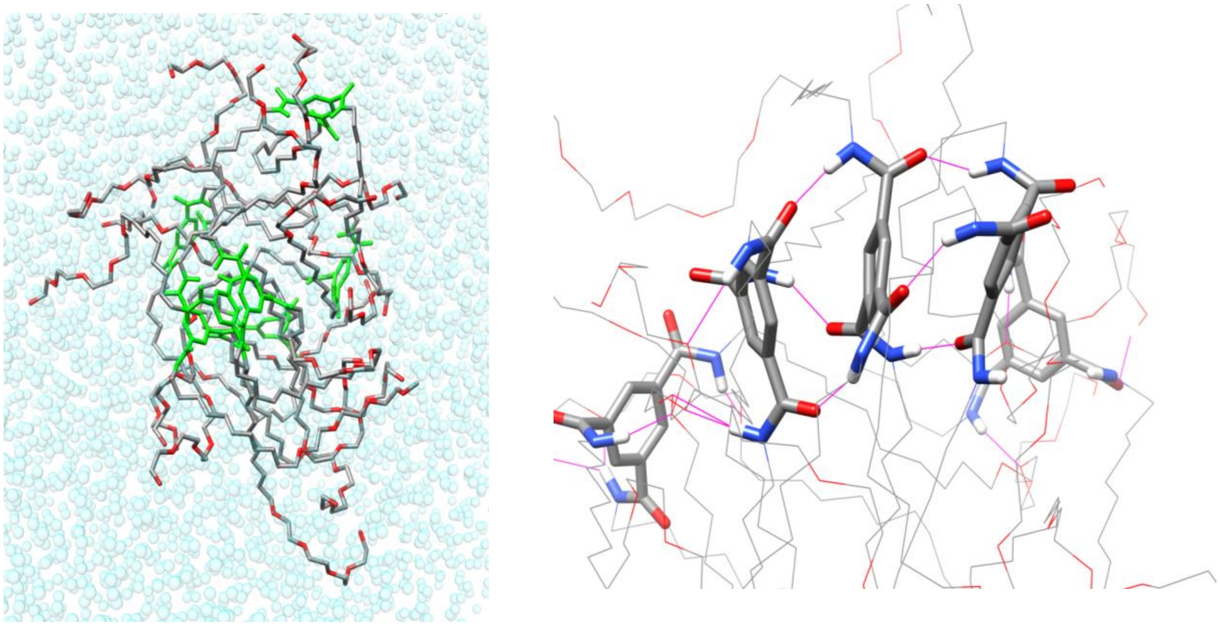

Supplementary Figure 26: Final snapshot of the MD simulation of seven BTAs initially molecularly dissolved in water. During the MD simulation the BTAs aggregate in solution. After ≈150 ns, three BTAs (in stick) formed a stable, hydrogen bound stack that persisted for the last ≈250 ns of the simulation (see SI movie). Hydrogen bonds are colored in pink. Details of these simulations will be presented in a subsequent publication, but are shown here to support the robustness of the methods employed.

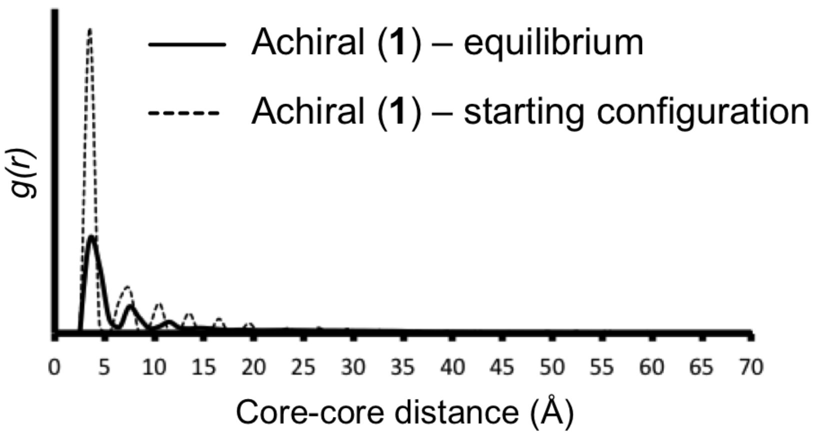

Supplementary Figure 27: Radial distribution functions *g(r)* of the BTA cores (representative of the core-core stacking distances) obtained from the MD simulation of the achiral (1) fiber.

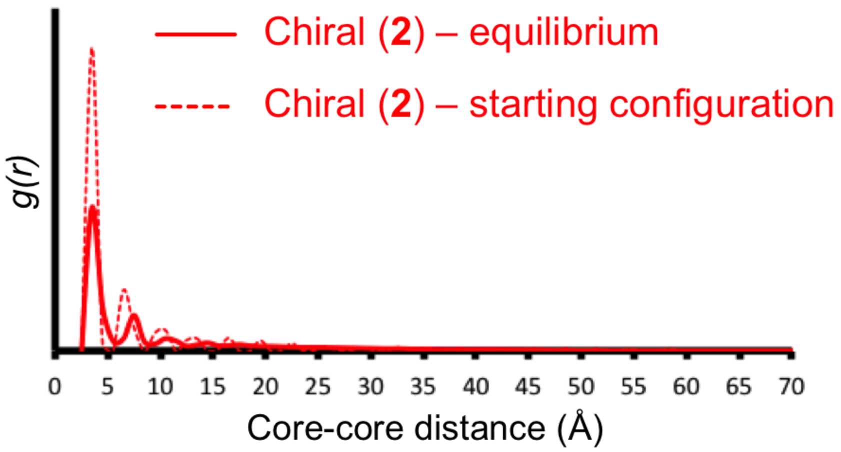

Supplementary Figure 28: Radial distribution functions *g(r)* of the BTA cores (representative of the core-core stacking distances) obtained from the MD simulation of the achiral (2) fiber.

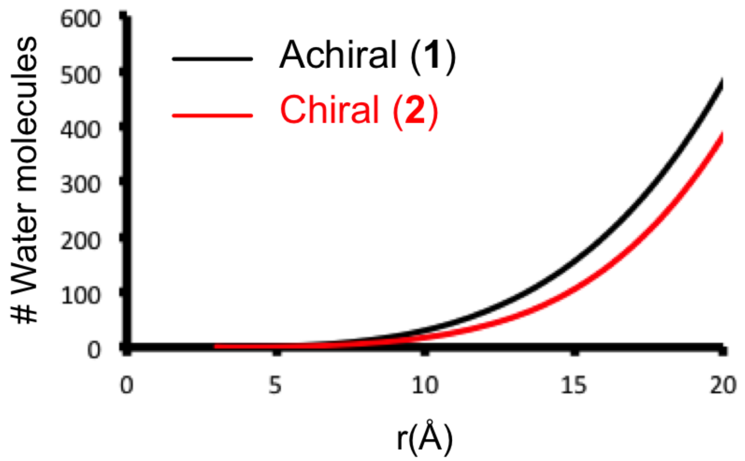

Supplementary Figure 29: Number of water molecules at variance of the distance from center of fiber

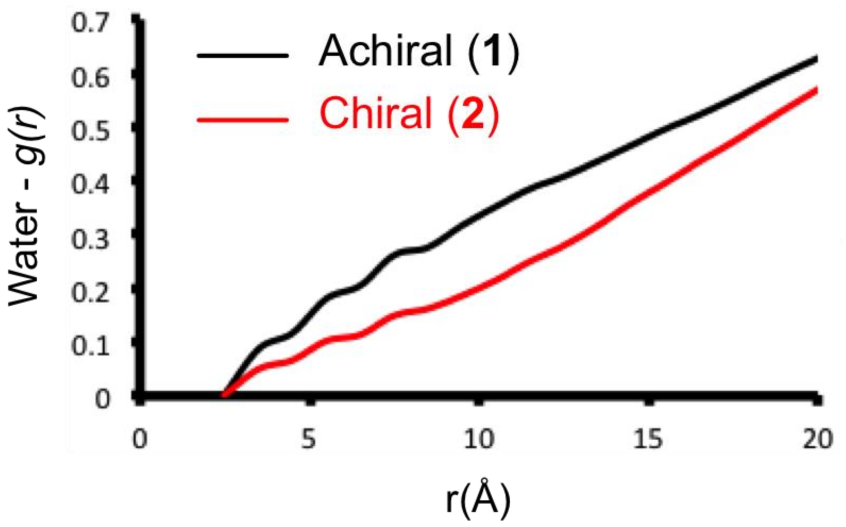

Supplementary Figure 30: Radial distribution functions *g(r)* of water molecules at variance of the distance from center of fiber

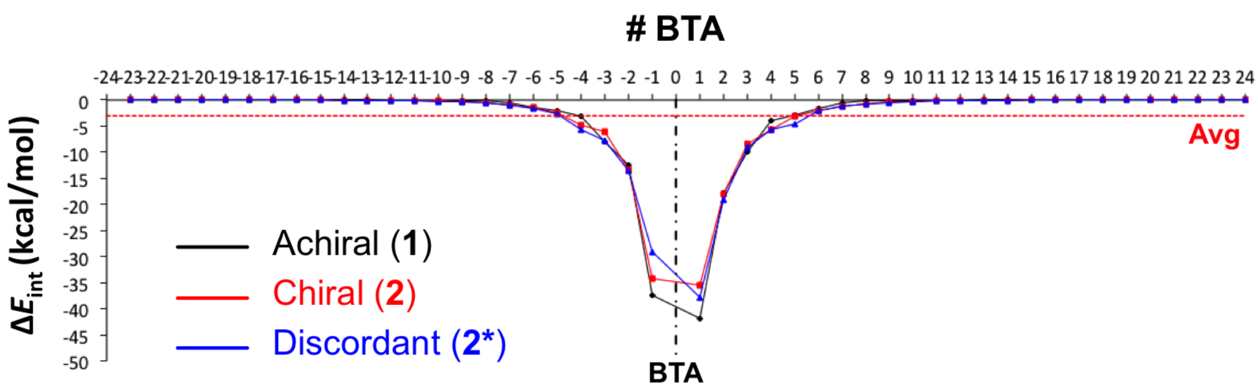

Supplementary Figure 31: Distribution of the pairwise (ΔE_{int}) interaction energies of each BTA with the others in the fiber at variance of their position. Data are averaged over the equilibrated phase MD trajectories (the last 100 ns of the MD simulations). Data show that each BTA interacts strongly with the closest neighbor, and the BTA-BTA interaction rapidly decreases for increasing distance from the BTA (becoming negligible after the ≈8th neighbor). The average ΔE_{int} (≈-3.0 kcal/mol for all cases) is represented as a dotted red line.

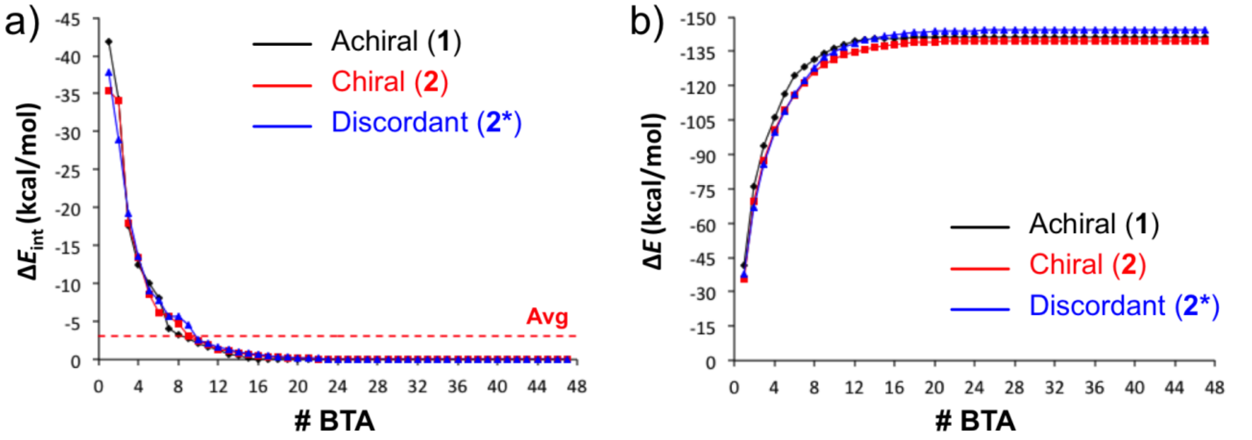

Supplementary Figure 32: Distribution of the self-assembly energy for the BTAs along the fiber. (a) The pairwise ΔE_{int} data (same as reported in Figure S24) at variance of the BTA neighbor number. (b) Cumulative ΔE self-assembly energy (ΔE) data calculated accounting for an increasing number of neighbor BTAs in the fiber at increasing distance from the BTAs. The ΔE data saturates after ≈16-20 neighbor BTAs (≈8-10 per-side), demonstrating that our model containing 48 BTA, where each BTA has 23.5 neighbors, is, from the interaction energies point of view, representative of the fibers bulk. The maximum plateau value of the ΔE divided for the total number of BTA neighbors gives the average per-BTA ΔE values (≈3.0 kcal/mol – red dotted line in panel (a)) reported in Figure 4 and Figure 5 in the main paper.

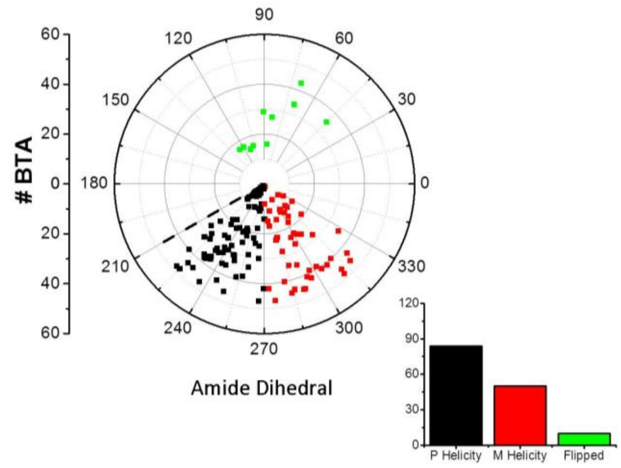

Supplementary Figure 33: Radial distribution of amide dihedrals calculated from the last 100ns of MD simulation for the discordant (2*) fiber. Note that this fiber does retain some of its initial *P*-helicity.

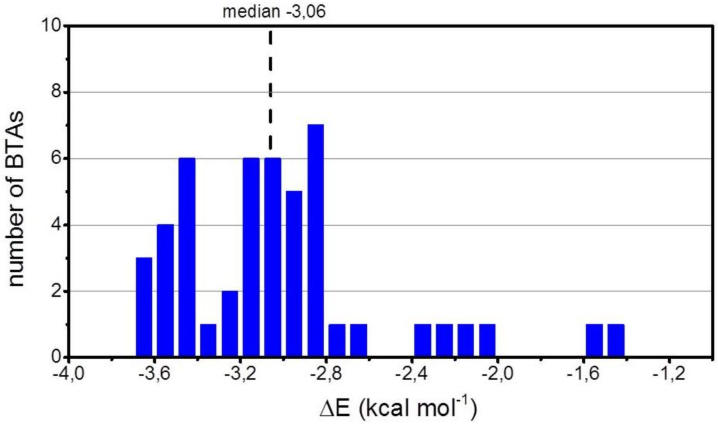

Supplementary Figure 34: Histogram of individual self-assembly energies (Δ*E*) for BTAs within the discordant (2*) fiber.

Infrared Spectroscopy

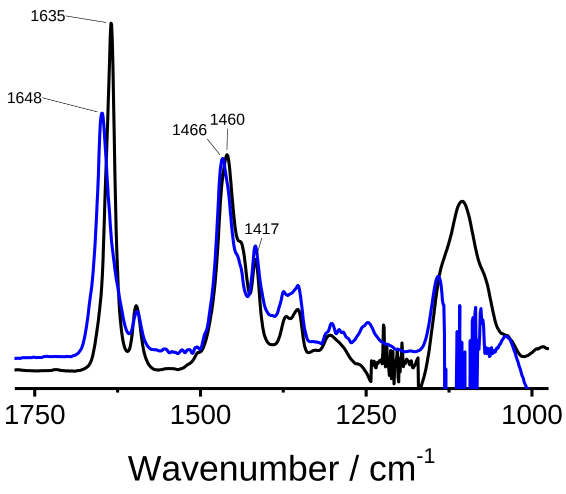

Supplementary Figure 35: IR spectrum of a solution of 1 (c = 50 mg/ml) at room temperature in D2O (black) displaying a carbonyl stretch vibration indicative for stacked hydrogen bonding and MeOD (blue) displaying a carbonyl stretch vibration associated with molecular dissolution (measured using a CaF2 cell with pathlength 0.05 mm).

Supplementary Methods

Detailed synthetic Procedures

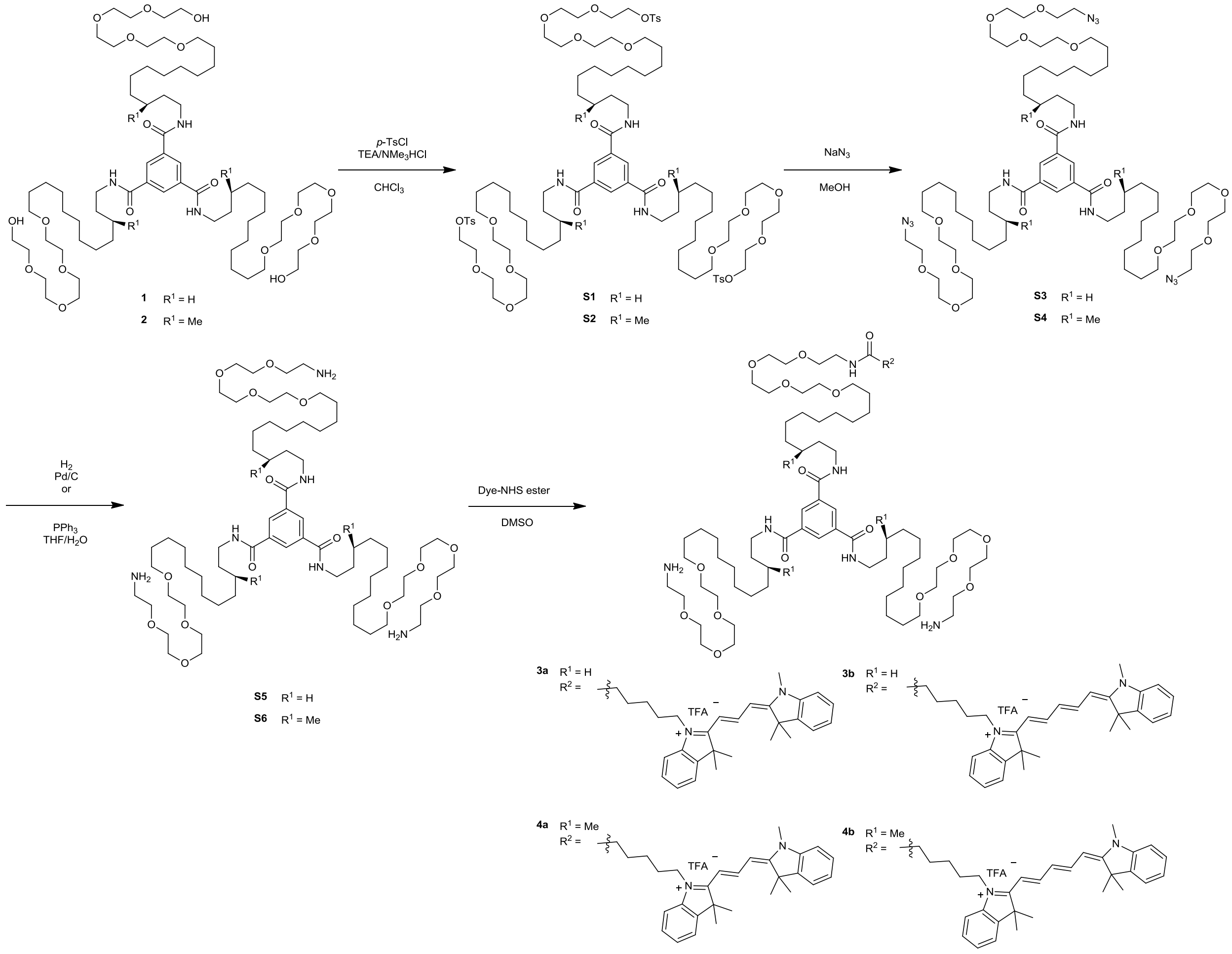

1,1',1''-(benzene-1,3,5-triyl)tris(1-oxo-15,18,21,24-tetraoxa-2-azahexacosane-26,1-diyl) tris(4-methylbenzenesulfonate) (S1). A 5 mL round-bottomed flask was charged with BTA **1**[1] (25 mg, 19.4 µmol), *para*-toluenesulfonic acid chloride (22 mg, 0.12 mmol), and trimethylamine hydrochloride (3 mg, 0.03 mmol) in $CHCl_3$ (Volume: 2 ml) to give a colorless solution. Then, 3 drops of TEA were added to the reaction mixture and the reaction was allowed to run over 24 hours. The reaction mixture was purified *via* column chromatography to yield the tri-tosylated BTA **S1** as a colorless oil (30 mg, 16.9 µmol, 87% yield). TLC ($CHCl_3$:MeOH, 96:4 v/v): $R_f \approx 0.35$; ^{1}H NMR (400 MHz, $CDCl_3$): δ 8.35 (s, 3H), 7.78 (d, J = 8.0 Hz, 6H), 7.33 (d, J = 8.0 Hz, 6H), 6.60 (t, J = 5.6 Hz, 3H), 4.14 (t, J = 4.9 Hz, 6H), 3.70–3.35 (m, 54H), 2.44 (s, 9H), 1.70–1.05 (m, 57H); ^{13}C NMR (100 MHz, $CDCl_3$) δ 165.8, 144.9, 135.4, 133.1, 130.0, 128.2, 128.1, 71.7, 70.9, 70.8, 70.7, 70.6, 70.2, 69.4, 68.8, 40.5, 29.7, 29.7, 29.6, 29.6, 29.6, 29.4, 27.1, 26.2, 21.8; MS (ESI, m/z): $[M+H]^+$ calcd. for $C_{90}H_{148}N_3O_{24}S_3$ 1751.96; found, 1752.18.

(5*S*,5'*S*,5''*S*)-1,1',1''-(benzene-1,3,5-triyl)tris(5-methyl-1-oxo-15,18,21,24-tetraoxa-2-azahexacosane-26,1-diyl) tris(4-methylbenzenesulfonate) (S2). A 5 mL round-bottomed flask was charged with chiral

BTA **2**[1] (14 mg, 10.5 µmol), *para*-toluenesulfonic acid chloride (30 mg, 0.16 mmol), and trimethylamine hydrochloride (3 mg, 0.03 mmol) in $CHCl_3$ (Volume: 2 ml) to give a colorless solution. Then, 3 drops of TEA were added to the reaction mixture and the reaction was allowed to run over 48 hours; at 24 hours another equivalent of trimethylamine hydrochloride (3 mg, 0.03 mmol) was added to the reaction. The reaction mixture was purified *via* column chromatography to yield the tri-tosylated BTA **S2** as a colorless oil (12 mg, 6.7 µmol, 64% yield). TLC ($CHCl_3$:MeOH, 96:4 v/v): $R_f \approx 0.35$; ^1H NMR (400 MHz, $CDCl_3$) δ 8.35 (s, 3H), 7.78 (d, *J* = 8.0 Hz, 6H), 7.33 (d, *J* = 8.0 Hz, 6H), 6.59 (t, *J* = 5.6 Hz, 3H), 4.13 (t, *J* = 4.9 Hz, 6H), 3.70–3.35 (m, 54H), 2.44 (s, 9H), 1.70–1.05 (m, 57H), 0.93 (d, *J* = 6.4 Hz, 9H); ^{13}C NMR (100 MHz, $CDCl_3$) δ 165.8, 144.9, 135.4, 133.1, 130.0, 128.2, 128.1, 71.7, 70.9, 70.8, 70.7, 70.6, 70.2, 69.4, 68.8, 38.6, 36.9, 36.7, 30.8, 30.0, 29.7, 29.7, 29.6, 27.0, 26.2, 21.8, 19.7; MS (ESI, m/z): $[M+H]^+$ calcd. for $C_{93}H_{154}N_3O_{24}S_3$, 1793.01; found, 1792.78.

***N*[1],*N*[3],*N*[5]-tris(1-azido-3,6,9,12-tetraoxatetracosan-24-yl)benzene-1,3,5-tricarboxamide (S3).** In a 5 mL sealed vial tri-tosylated BTA **S1** (20 mg, 11.1 µmol) and sodium azide (30 mg, 0,616 mmol) were suspended in methanol (1.5 ml) to give a colorless suspension. The suspension was then heated to 60°C over 16 hours, monitoring reaction conversion *via* TLC. Upon completion, MeOH was removed, the residue suspended in $CHCl_3$ and then purified *via* column chromatography yielding the tri-azide BTA **S3** as a colorless oil (15.6 mg, 11.1 µmol, quant.). Characterization matches literature.[2] TLC ($CHCl_3$:MeOH, 94:6 v/v): $R_f \approx 0.3$; ^1H NMR (400 MHz, $CDCl_3$) δ 8.36 (s, 3H), 6.65 (t, *J* = 5.5 Hz, 3H), 3.70–3.45 (m, 48H), 3.45–3.30 (m, 12H), 1.70–1.15 (m, 57H).

***N*[1],*N*[3],*N*[5]-tris((*S*)-1-azido-22-methyl-3,6,9,12-tetraoxatetracosan-24-yl)benzene-1,3,5-tricarboxamide (S4)**. In a 5 mL sealed vial tri-tosylated BTA **S2** (11 mg, 6.1 µmol) and sodium azide (20 mg, 0,308 mmol) were suspended in methanol (1 ml) to give a colorless suspension. The suspension was then heated to 60°C over 48 hours, monitoring reaction conversion *via* TLC. Upon completion, MeOH was removed, the residue suspended in $CHCl_3$ and then purified *via* column chromatography yielding the tri-azide BTA S**4** as a colorless oil (8.5 mg, 6.1 µmol, quant.). TLC ($CHCl_3$:MeOH, 94:6 v/v): $R_f \approx 0.3$; ^1H NMR (400 MHz, $CDCl_3$) δ 8.26 (s, 3H), 6.55 (t, *J* = 5.5 Hz, 3H), 3.70–3.45 (m, 48H), 3.42 (t, *J* = 6.8 Hz, 6H), 3.37 (t, *J* = 5.1 Hz, 6H), 1.70–1.05 (m, 57H), 0.94 (d, *J* = 6.4 Hz, 9H); ^{13}C NMR (100 MHz, $CDCl_3$) δ 165.7, 135.4, 128.2, 71.7, 70.8, 70.8, 70.8, 70.2, 70.2, 50.8, 38.6, 36.9, 36.8, 30.8, 30.0, 29.7, 29.7, 29.6, 27.0, 26.2, 19.7; MS (ESI, m/z) $[M+H]^+$ calcd. for $C_{72}H_{134}N_{12}O_{15}$, 1406.00; found, 1406.00.

N*[1],*N*[3],*N*[5]-tris(1-amino-3,6,9,12-tetraoxatetracosan-24-yl)benzene-1,3,5-tricarboxamide (S5)**. In a 5 mL round-bottomed flask tri-azide BTA **S3** (15 mg, 11 µmol) and triphenyl phosphine (5 mg, 19 µmol) were dissolved in 2 mL of THF. After stirring at room temperature overnight, 500 µL of H_2O was added and the reaction was allowed to stir at room temperature over a second night. The reaction could be monitored by TLC (5% MeOH/$CHCl_3$). After completion of hydrolysis, the reaction mixture was loaded directly on a silica column. Triphenylphospine and its oxide could be removed by eluting with 5% MeOH/$CHCl_3$, while the product only moved upon addition of *i*-$PrNH_2$ (1%), yielding **S5** as a colorless solid (12 mg, 9 µmol, 81%). Characterization matches literature.[2] ***It is worth noting that this is the preferred method of reduction, as it prevents unwanted side products and is much cleaner. TLC

($CHCl_3$:MeOH:*i*-$PrNH_2$, 90:8:2 v/v): $R_f \approx 0.3$; ^{1}H NMR (400 MHz, $CDCl_3$) δ 8.38 (s, 3H), 6.87 (t, J = 5.7 Hz, 3H), 3.70–3.45 (m, 48H), 3.50–3.35 (m, 18H), 1.58 (m, 12H), 1.40–1.20 (m, 57H).

***N*^{1},*N*^{3},*N*^{5}-tris((*S*)-1-amino-22-methyl-3,6,9,12-tetraoxatetracosan-24-yl)benzene-1,3,5-tricarboxamide (S6)**. In a 5 mL round-bottomed flask tri-azide BTA **S4** (8 mg, 5.69 µmol), palladium on carbon (5 mg, 4.70 µmol), and ammonium hydroxide (120 µl, 3.08 mmol) were dissolved in methanol (Volume: 1 mL) to give a black suspension. The solution was then purged with H_2 and stirred overnight under a positive H_2 atmosphere (balloon). The suspension was then filtered and washed multiple times with MeOH, followed by $CHCl_3$. The material was purified via column chromatography, yielding **6** as a waxy solid (6 mg, 4.52 µmol, 79% yield). TLC ($CHCl_3$:MeOH:*i*-$PrNH_2$, 90:8:2 v/v): $R_f \approx 0.3$; ^{1}H NMR (400 MHz, $CDCl_3$) δ 8.38 (s, 3H), 6.82 (t, J = 5.7 Hz, 3H), 3.70–3.55 (m, 42H), 3.49 (t, J = 5.1 Hz, 6H), 3.42 (t, J = 6.8 Hz, 6H), 2.84 (t, J = 5.2 Hz, 6H), 1.70–1.05 (m, 57H), 0.94 (d, J = 6.5 Hz, 9H); ^{13}C NMR (100 MHz, $CDCl_3$) δ 165.9, 135.4, 128.3, 77.4, 73.4, 71.7, 70.8, 70.7, 70.7, 70.4, 70.2, 41.8, 38.6, 36.9, 36.7, 30.8, 29.9, 29.7, 29.7, 29.5, 27.0, 26.2, 19.7; MS (MALDI, m/z) $[M+H]^+$ calcd. for $C_{72}H_{139}N_6O_{15}$, 1328.03; found, 1328.03.

Supplementary References

1. Leenders, C. M. A. *et al.* Supramolecular polymerization in water harnessing both hydrophobic effects and hydrogen bond formation. *Chem. Commun.* **49,** 1963–1965 (2013).

2. Albertazzi, L. *et al.* Spatiotemporal control and superselectivity in supramolecular polymers using multivalency. *Proc. Natl. Acad. Sci. U. S. A.* **110,** 12203–12208 (2013).
